# A novel injectable fibromodulin‐releasing granular hydrogel for tendon healing and functional recovery

**DOI:** 10.1002/btm2.10355

**Published:** 2022-07-14

**Authors:** Xue Xu, Yulong Zhang, Pin Ha, Yao Chen, Chenshuang Li, Emily Yen, Yuxing Bai, Renji Chen, Benjamin M. Wu, Andrew Da Lio, Kang Ting, Chia Soo, Zhong Zheng

**Affiliations:** ^1^ Department of Oral and Maxillofacial Plastic and Traumatic Surgery Beijing Stomatological Hospital of Capital Medical University Beijing China; ^2^ Division of Plastic and Reconstructive Surgery David Geffen School of Medicine, University of California Los Angeles California USA; ^3^ Division of Growth and Development School of Dentistry, University of California Los Angeles California USA; ^4^ School of Dentistry University of California Los Angeles California USA; ^5^ Department of Orthodontics School of Dental Medicine, University of Pennsylvania Philadelphia Pennsylvania USA; ^6^ Arcadia High School Arcadia California USA; ^7^ Department of Orthodontics Beijing Stomatological Hospital of Capital Medical University Beijing China; ^8^ Forsyth Research Institute Harvard University Cambridge Massachusetts USA; ^9^ Samueli School of Engineering University of California Los Angeles California USA; ^10^ Division of Plastic and Reconstructive Surgery, Department of Orthopaedic Surgery The Orthopaedic Hospital Research Center, University of California Los Angeles California USA

**Keywords:** fibromodulin, functional reconstruction, granular hydrogel, hydrogel microparticles, tendon wound healing

## Abstract

A crucial component of the musculoskeletal system, the tendon is one of the most commonly injured tissues in the body. In severe cases, the ruptured tendon leads to permanent dysfunction. Although many efforts have been devoted to seeking a safe and efficient treatment for enhancing tendon healing, currently existing treatments have not yet achieved a major clinical improvement. Here, an injectable granular hyaluronic acid (gHA)‐hydrogel is engineered to deliver fibromodulin (FMOD)—a bioactive extracellular matrix (ECM) that enhances tenocyte mobility and optimizes the surrounding ECM assembly for tendon healing. The FMOD‐releasing granular HA (FMOD/gHA)‐hydrogel exhibits unique characteristics that are desired for both patients and health providers, such as permitting a microinvasive application and displaying a burst‐to‐sustained two‐phase release of FMOD, which leads to a prompt FMOD delivery followed by a constant dose‐maintaining period. Importantly, the generated FMOD‐releasing granular HA hydrogel significantly augmented tendon‐healing in a fully‐ruptured rat's Achilles tendon model histologically, mechanically, and functionally. Particularly, the breaking strength of the wounded tendon and the gait performance of treated rats returns to the same normal level as the healthy controls. In summary, a novel effective FMOD/gHA‐hydrogel is developed in response to the urgent demand for promoting tendon healing.

## 1 INTRODUCTION

In the musculoskeletal system, tendons transmit the mechanical force from skeletal muscle to the bone and facilitate movement due to their stiff and viscoelastic nature.^1^, ^2^ About 75% of total tendon ruptures are related to sports activities and vice versa 30%–50% of sporting injuries are related to a tendon injury,^3^, ^4^, ^5^, ^6^, ^7^, ^8^ resulting in a diversity of complications and even disability,^9^ which significantly decrease the patients' quality of life and place a tremendous financial burden on individuals and the health system.^10^ Consequently, approximately 300,000 tendon procedures are performed in the United States annually,^11^ and over 30 billion US dollars and 115 billion euros are expended in treating tendon injuries.^12^

Tendon regeneration is extremely poor and inefficient due to its hypocellular and hypovascular nature.^6^, ^13^ A diversity of procedures, such as motion restriction, low‐energy laser therapy, shock‐wave treatment, cryotherapy, and injection of nonsteroidal anti‐inflammatory drugs and corticosteroids have been used to promote tendon healing.^6^, ^14^ These currently available treatments rarely gain a satisfactory prognosis with various sequelae because of poor tissue quality, inferior mechanical properties resulting from fibrotic scar tissue, and continuously reduced functionality.^6^, ^15^, ^16^, ^17^, ^18^ Efforts have also been devoted to growth factors for their use in promoting tendon healing.^2^, ^6^, ^19^, ^20^, ^21^ However, there are multiple growth factors produced and released during the tendon healing process and the variability of the “growth factor/cytokine cocktail” reported in the publications are inconsistent in their efficacy.^6^ Combining numerous bioactive molecules as a combo therapy also posts an additional allergy/rejection risk and significantly raises the regulatory bars for their clinical approval. Therefore, an impetus exists for seeking novel strategies that can initiate endogenous tissue repair, accelerate tendon wound healing, and direct the healing process toward reconstructing the physiologically normal tendons with an uncomplicated bioactive molecule recipe.^6^

It is worth noting that the composition and fibrillar structure of the tendon extracellular matrix (ECM) predominantly determine the tendon's force‐transmitting function.^6^, ^22^, ^23^ As both structural components and signal transduction modulators, ECM is gaining more and more attention recently for wound healing management. For example, fibromodulin (FMOD) is a 59‐kD small leucine‐rich proteoglycan (SLRP) that regulates tendon collagen fibrillogenesis during the entire tendon developing period.^24^ FMOD binds to the growing fibrils and modifies covalent cross‐linking of type I collagen mechanically by regulating lysyl oxidase (LOX) activity on the c‐telopeptide lysine,^25^, ^26^ resulting in stable trivalent cross‐linking and thus enhancing collagen interconnectivity and fibril stability.^26^, ^27^ Consequently, FMOD deficiency decreases tendon stiffness and maximum load.^28^ Studies in multiple small and large animal cutaneous wound models also demonstrate that FMOD promotes fibroblast migration to accelerate wound healing, orchestrates collagen fibrogenesis to reduce scar formation, and enhances tensile strength reestablishment.^26^, ^29^, ^30^, ^31^, ^32^ Considering that tenocytes are the major cell type residents in the tendon compartment that are defined as “specialized” fibroblasts responsible for ECM synthesis and tendon regeneration predominantly,^33^, ^34^, ^35^ the advantageous pro‐healing potency of FMOD in skin wounds is highly likely to be duplicated in the injured tendons.

Safely and effectively delivering the pharmacological agent, is another prerequisite for an effective therapeutic strategy. In the last two decades, the usage of hydrogels has expanded dramatically, particularly for a controlled drug release to achieve better activity after application.^36^ Hydrogels are hydrophilic polymer networks prepared by physical or chemical cross‐linking of synthetic and natural hydrophilic polymers with a unique three‐dimensional (3D) structure that can absorb large amounts of water or biological fluids.^36^ In general, hydrogels swell readily and remain soft and rubbery, resembling the properties of living tissues.^37^ Controlling the imbibition degree, cross‐linking extent, and the biodegradation rate of hydrogels can achieve a desired drug release schedule.^37^ For instance, its aqueous solubility allows for the modification of hyaluronic acid (HA) into various hydrogel systems with porous and 3D structures as the vehicle for growth factors, morphogens, and stem cells.^38^, ^39^, ^40^ Moreover, HA is highly biocompatible with tenocytes without interrupting their function for tendon repair,^39^ suggesting HA hydrogel is a suitable raw material as an FMOD‐delivery vehicle. However, the conventional HA hydrogel is not injectable and hardly fills in the small and/or irregular‐shaped wounded sites, which is not desirable for clinical application and reduces the efficacy. On the other hand, photopolymerization technology provides the opportunity to cross‐link the hydrogel after the polymer (such as HA) fills in the injured area, while the photopolymerization inducers may also interact with the contained bio‐active molecule,^41^ and thus diminish the pro‐healing potency. Therefore, a novel form of delivery vehicle is needed.

In this study, the pro‐healing effects of FMOD were first confirmed on tenocytes in vitro, and an injectable, FMOD‐releasing granular HA hydrogel (FMOD/gHA‐hydrogel) was developed for in vivo application. A rat's Achilles tendon injury model was used to explore the potential benefits of the engineered FMOD/gHA‐hydrogel on tendon healing. The animals were evaluated histologically, mechanically, and functionally for a comprehensive assessment. The ECM architecture, tensile strength, and gait performance of the rats with healing Achilles tendons were significantly improved in the group administrated with the FMOD/gHA‐hydrogel, which established a solid foundation for further pre‐clinical and clinical evaluations of the treatment potency of the FMOD/gHA‐hydrogel on tendon healing.

## 2 RESULTS

### 2.1 FMOD enhances tenocytes movement

**FIGURE 1:**
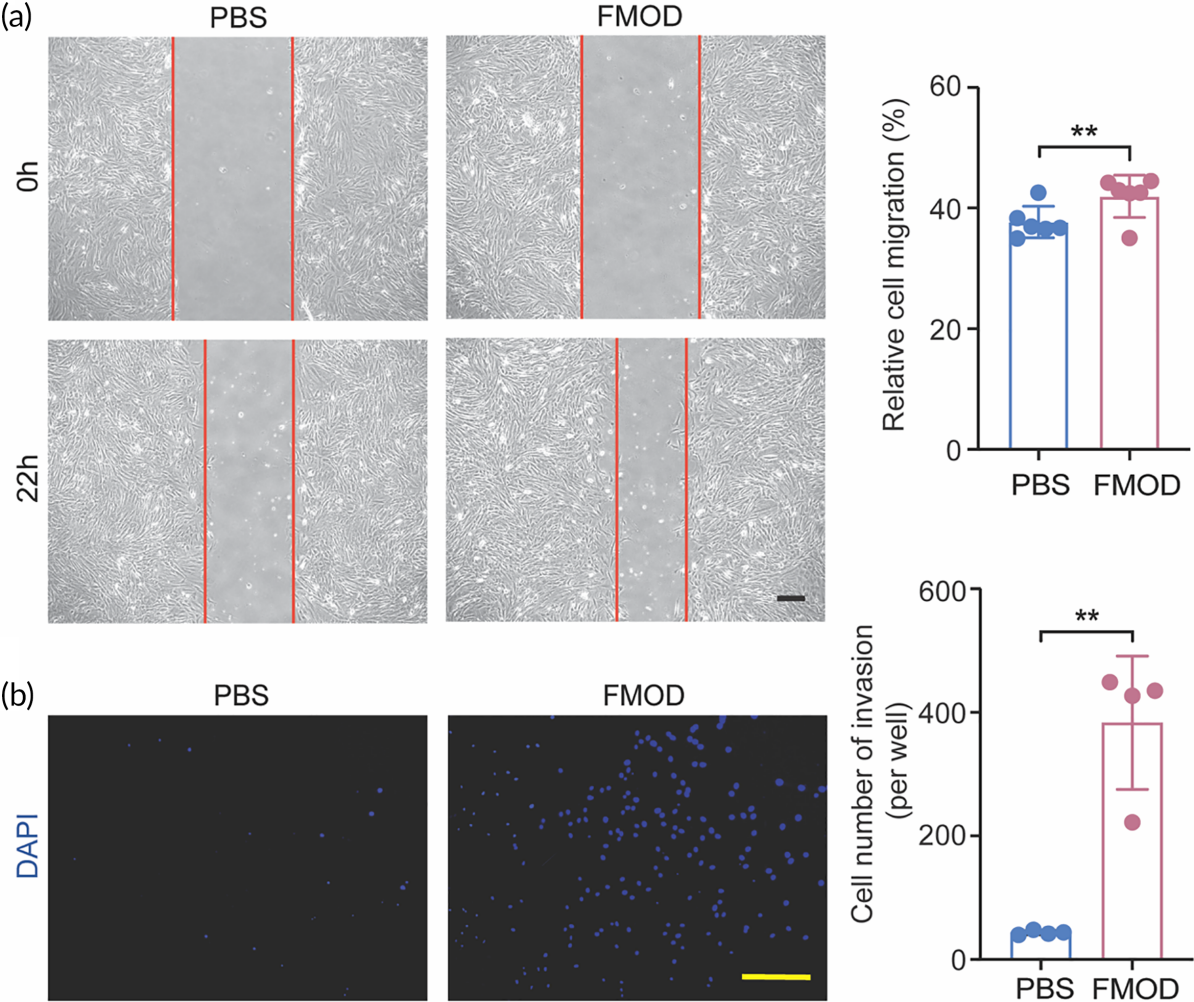
Fibromodulin (FMOD) improved tenocyte mobility. FMOD enhanced both tenocyte migration (a) and invasion (b). Scale bar = 200 μm (a) or 100 μm (b). Data are shown as mean ± SD, *n* = 6 (a) and 4 (b), respectively. Two‐sample *t*‐tests were used for statistical analyses. **p < 0.005.

As the predominant cell populations in tendons,^33^, ^34^ tenocytes are spindle‐shaped, fibroblast‐like cells embedded between collagen fibers and synthesize collagen fibrils during tendon development and healing.^6^, ^42^ Like dermal fibroblasts,^30^ tenocytes are insensitive to FMOD (constituted in PBS) regarding cell proliferation **(**Figure S1). The highest concentration of FMOD that does not prohibit cell proliferation was used in the following in vitro analyses. Surprisingly, although FMOD only slightly enhanced tenocyte migration by 4.26% (Figure 1a), it significantly promoted tenocyte invasion through the collagen matrices by around 8.8‐fold (Figure 1b).

**FIGURE 2:**
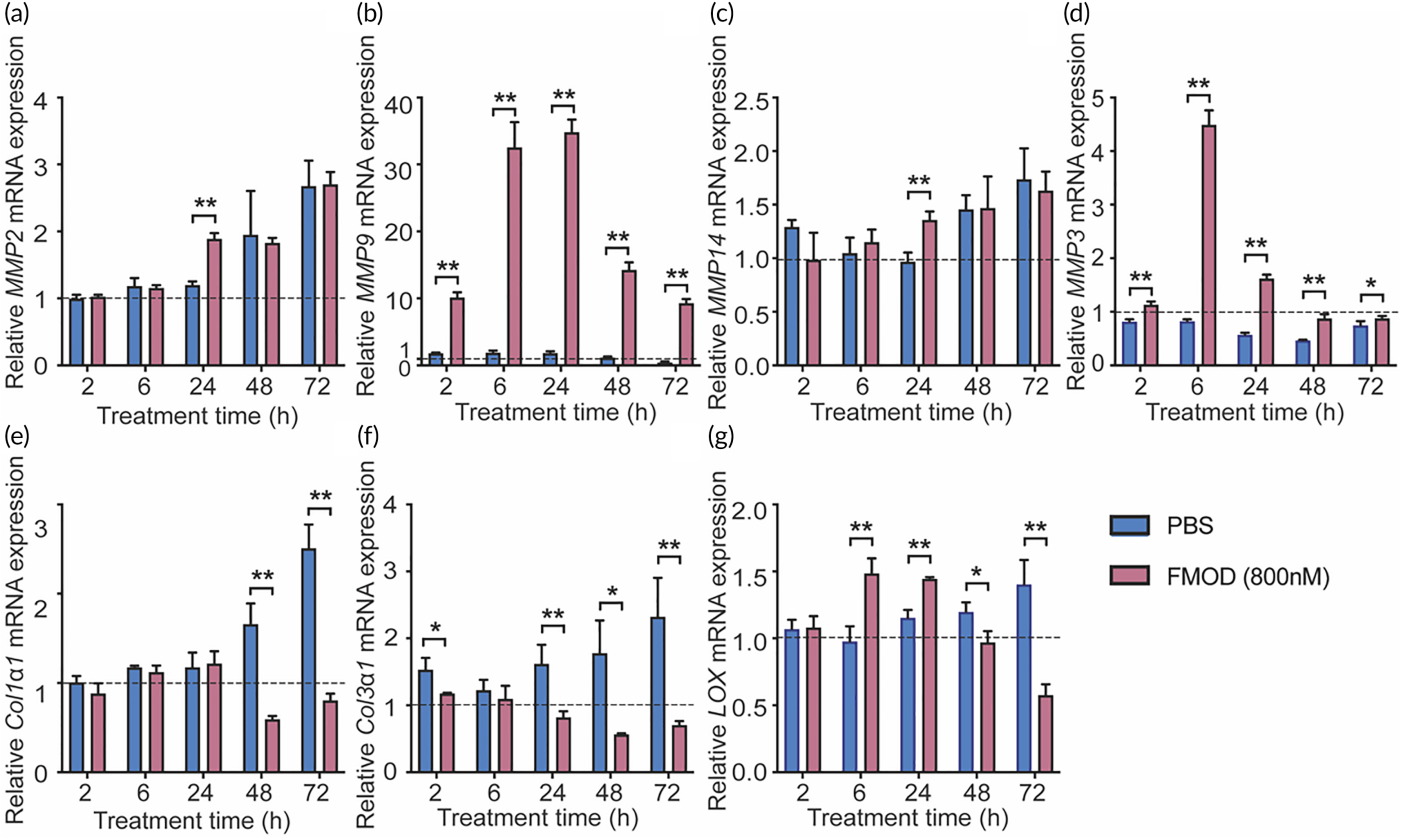
Fibromodulin (FMOD) modulated tenocyte gene expression. Relative expressions of *MMP2* (a), *MMP9* (b), *MMP14* (c), *MMP3* (d), *Col1α1* (e), *Col3α1* (f), and *LOX* (g) against *GAPDH* were normalized to the initial pre‐treatment values. Data are shown as mean ± SD, *n* = 3. Two‐sample *t*‐tests were used for statistical analysis. *p < 0.05; **p < 0.005.

Among matrix‐degrading proteinases, matrix metalloproteinases (MMPs) are a family of neutral endopeptidases with a broad proteolytic capability^43^ that are essential for tissue repair initiation,^44^, ^45^, ^46^ including tendon healing.^47^ Specifically, MMP2 and MMP9 are correlated with tenocyte migration and invasion.^48^, ^49^ FMOD slightly enhanced *MMP2* expression 24‐h post‐treatment (Figure 2a), but significantly stimulated MMP9 expression at transcriptional and translational levels during the entire 72‐h experimental period (Figure 2b and Figure S2A‐B).

### 2.2 FMOD alters tendon extracellular molecule expression levels

Since MMPs' activities directly determine the integrity of the ECM, upregulating MMPs is also a typical indicator for ECM reassembly,^45^ which is particularly true for MMP2 and MMP9 during tendon wound healing.^50^ MMP3 and MMP14 are the other two MMP members participating in matrix degradation and remodeling throughout the healing process.^50^ As MMP14 is vital for MMP2 activation and has MMP2‐dependent roles in connective‐tissue remodeling,^51^, ^52^, ^53^ it is no surprise that FMOD only slightly and temporarily induced tenocyte *MMP14* expression 24‐h post‐treatment (Figure 2c), mirroring the expression pattern of *MMP2* (Figure 2a). In contrast, MMP3, which is thought to play a more prevailing role in tendon ECM remodeling and tissue repair,^46^, ^54^ was significantly upregulated by FMOD on both mRNA and protein levels (Figure 2d, Figure S2A, C).

Moreover, FMOD significantly downregulated *Col1α1* and *Col3α1*, which encode type I and III collagen, respectively (Figure 2e, f) along with the upregulation of MMP3 and MMP9. In addition, FMOD expedited tenocyte LOX upregulation (Figure 2g and Figure S2A, D).

### 2.3 A novel granular HA hydrogel for FMOD delivery is generated and characterized

It is worth noting that the tough, high‐tensile‐strength ECM structure, and low vascularity nature of tendon make drug application more cumbersome since the local intratendinous injection may not spread the bioactive component throughout the entire injured site as desired. Thus, a suitable delivery vehicle manufactured from a biocompatible, biodegradable material that is also highly retained in water and has enough viscosity to stick on the tissue surface, such as HA, is necessary.

**FIGURE 3:**
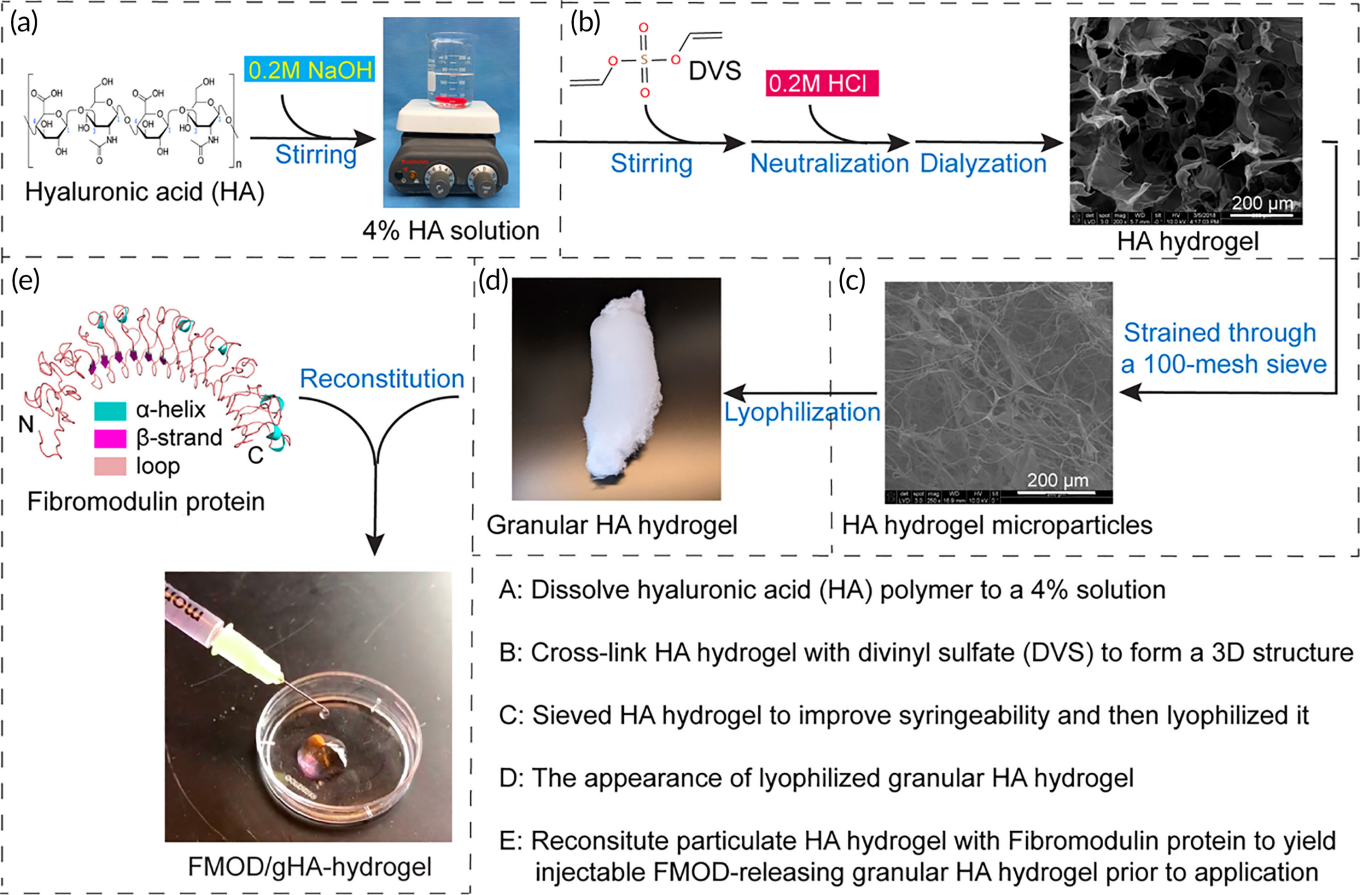
Fibromodulin (FMOD)‐releasing granular HA hydrogel was engineered based on a delicately designed blueprint. Four percent high molecular weight (HMW) HA solution (a) was cross‐linked with DVS (b). The formed bulk HA hydrogel was mechanically fragmented to fabricate HA hydrogel microparticles (HMPs) with a fine and fiber‐like porous structure (c), and then lyophilized to pack into granular HA hydrogel (d). The granular HA hydrogel was reconstituted with FMOD protein prior to use (e).

Since high molecular weight (HMW; Mw > 1000 kDa) HA exhibits anti‐inflammatory effects^55^ and is able to minimize the molecular weight loss during storage and application,^56^ HMW HA was chosen in this study and cross‐linked via divinyl sulfone (DVS) to form a porous HA hydrogel (Figure 3a,b). Besides, DVS‐cross‐linked HA hydrogels have been approved for human use for a divert of applications^57^, ^58^ which significantly decrease the safety concerns. However, bulk hydrogels are not always suited for their intended applications, particularly when the injection is needed to minimize the procedures' invasiveness in the clinical setting.^59^ Therefore, hydrogel microparticles (HMPs), which exhibit several unique properties compared to bulk hydrogels, were generated in response. Noticeably, their small size (1–1000 μm) enables injections of HMPs through the needles.^59^ Accordingly, the cross‐linked HA hydrogel was processed with a mechanical fragmentation technique, and the fabricated HA HMPs displayed a fine and fiber‐like porous structure (Figure 3c). Then, lyophilization was used to pack HA HMPs,^60^ and the generated granular HA hydrogel (gHA‐hydrogel; Figure 3d) was stored at 4°C until reconstitution with the FMOD solution prior to injection. Due to the general injection‐favorable shear‐thinning property shared by granular hydrogels,^59^, ^61^, ^62^ the gHA‐hydrogel passed through a syringe needle effortlessly and returned to a viscoelastic state after being released from stress (Figure 3e and Video S1). This super microinvasive property is particularly important for gHA‐hydrogel as a drug delivery vehicle into dense tissues, such as tendons, where minimally invasive interventions are desired.

**FIGURE 4:**
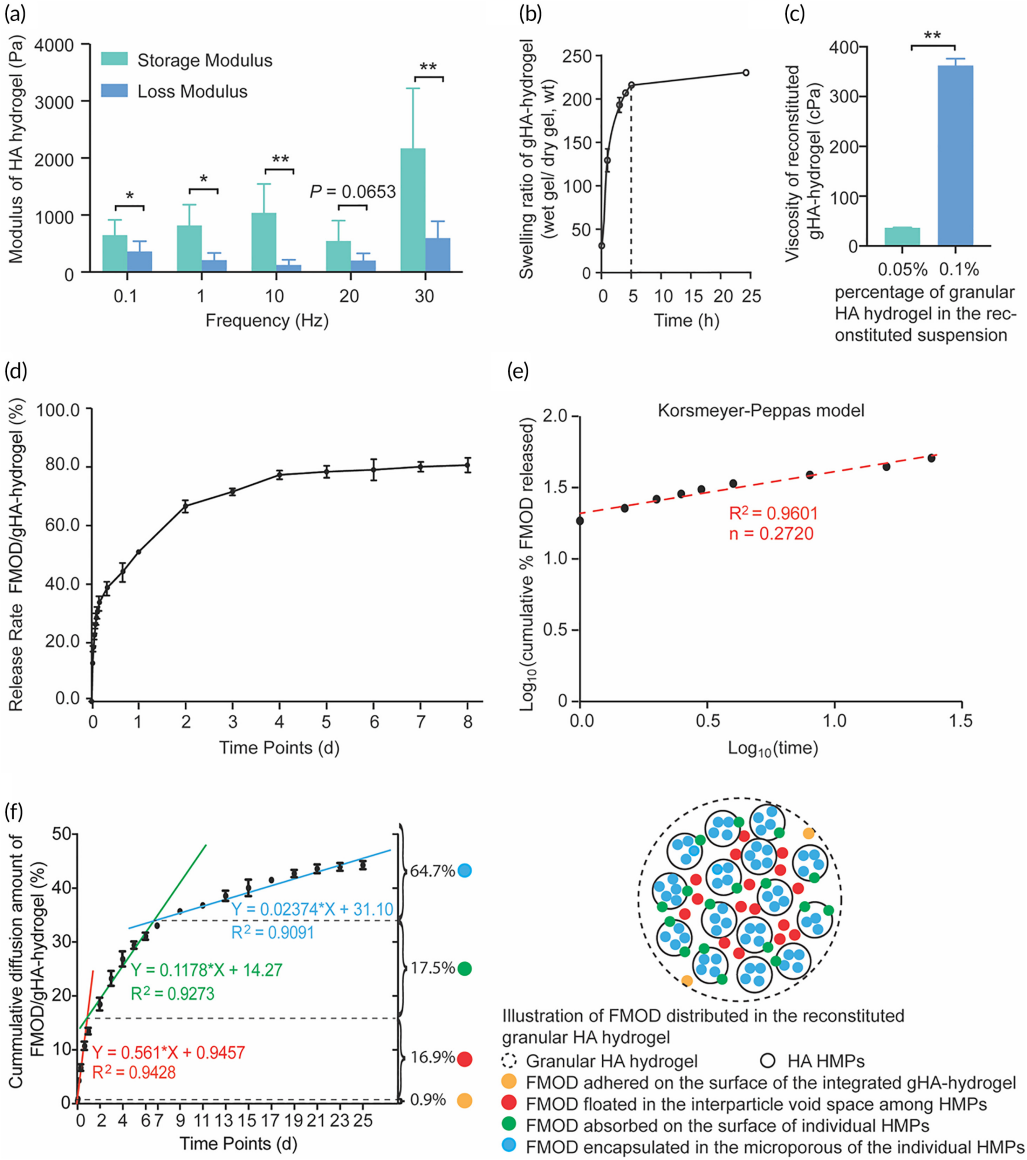
(FMOD)/gHA‐hydrogel was intensively characterized including storage and loss modulus measurements of cross‐linked HA‐hydrogel (a), the swelling ability of the gHA‐hydrogel (b), the viscosity of the reconstituted granular HA hydrogel at different gel concentrations (c), and the release profile of FMOD from the reconstituted gHA‐hydrogel (d), which fitted the Korsmeyer–Peppas kinetic model (e) and the distribution of FMOD in the reconstituted gHA‐hydrogel (f). Data are shown as mean ± SD, *n* = 8 (a), 3 (b–d, f). Mann–Whitney *U* tests (a) and two‐sample *t*‐tests (c) were used for statistical analyses. *p < 0.05; **p < 0.005.

The storage modulus of the cross‐linked HA hydrogel was much higher than its loss modulus (1.8–9.3 times, tan δ < 1) (Figure 4a), indicating the hydrogel was successfully crosslinked and is highly elastic, which agrees with tendons' viscoelastic nature. Echoing the previous statement that granular hydrogels can swell like bulk hydrogels,^59^, ^61^, ^62^ the generated gHA‐hydrogel could absorb 216 times of dry weight in water within 5 h, which extended to 233 times at 24 h (Figure 4b). This excellent water‐absorption ability of the gHA‐hydrogel represents its capability to minimize FMOD loss when exposed to wound exudate and blood and thus maintains the local dosage of applied FMOD. Meanwhile, the viscosity of the gHA‐hydrogel decreased significantly along with the increase of reconstituting solution (Figure 4c). Specifically, when the gHA‐hydrogel composited 0.1% (w/v) of the reconstituted suspensions, the viscosity was 362.5 cPa (Figure 4c), fairly enabling it to adhere and spread on various biological tissue surfaces and to fill arbitrarily shaped defects, such as seen in tendon injuries, without sacrificing its syringeability.

Because of their HMP/granular two‐scale matrix structure, granular hydrogels exhibit an interconnected microporous structure due to the interstitial pores that are formed when HMPs are packed together, resulting in numerous paths for an impregnated drug infiltration within the hydrogel structure.^59^, ^60^ To analyze the release kinetics of FMOD from the granular HA hydrogel, the release data was collected (Figure 4d) and fitted into multiple mathematical models (Figure S3 and Figure 4e). The fit agreement was achieved with the Korsmeyer–Peppas model (*R* ^2^ = 0.9601; Figure 4e), which describes drug release from a polymeric system.^63^ The diffusion exponent value “*n*” in the Korsmeyer–Peppas kinetic model is 0.2720 (<0.45), indicating a Quasi‐Fickian transport of FMOD from the granular HA hydrogel that was mainly controlled by the diffusing process.^63^, ^64^, ^65^ Using a one‐direction‐diffusing methodology,^66^, ^67^ the distribution of FMOD in the reconstituted gHA‐hydrogel was measured (Figure 4f). The hypothetical distribution was cataloged and estimated based on the different diffusion rates:^68^, ^69^ 0.9% adhered on the surface of the integrated gHA‐hydrogel, 16.9% floated in the interparticle void space among HPMs in the gHA‐hydrogel, 17.5% absorbed on the surface of individual HMPs, and 64.7% encapsulated in the nano−/micropores of the individual HMPs (Figure 4f). The complexity of FMOD distribution and interparticle friction among HMPs may explain the two‐phase release of FMOD from the gHA‐hydrogel was observed: a burst phase—the first 4 h in which ~33.8% of FMOD was released and a sustained phase—among 4 days in which ~43.5% of FMOD was released (Figure 4d). This burst‐to‐sustained two‐phase release capability indicates that the gHA‐hydrogel could firstly deliver FMOD to recruit tenocytes immediately and then maintain a constant dose of FMOD in a prolonged period with one single injection to reduce the injection‐related complications.

### 2.4 FMOD/gHA‐hydrogel significantly improved the histological outcomes of tendon wound healing

The Achilles tendon is the strongest and largest tendinous structure in the body and is one of the most commonly injured tissues. It is estimated to be responsible for about 50% of all sports injuries.^4^, ^70^, ^71^ Moreover, the incidence of Achilles tendon rupture increases every year.^5^ Thus, a rat Achilles tendon injury model was recruited in this study to assess the effectiveness of FMOD/gHA‐hydrogel, in which the adult rat's right Achilles tendon was completely transected to mimic the most severe injury scenario (Figure S4).

**FIGURE 5:**
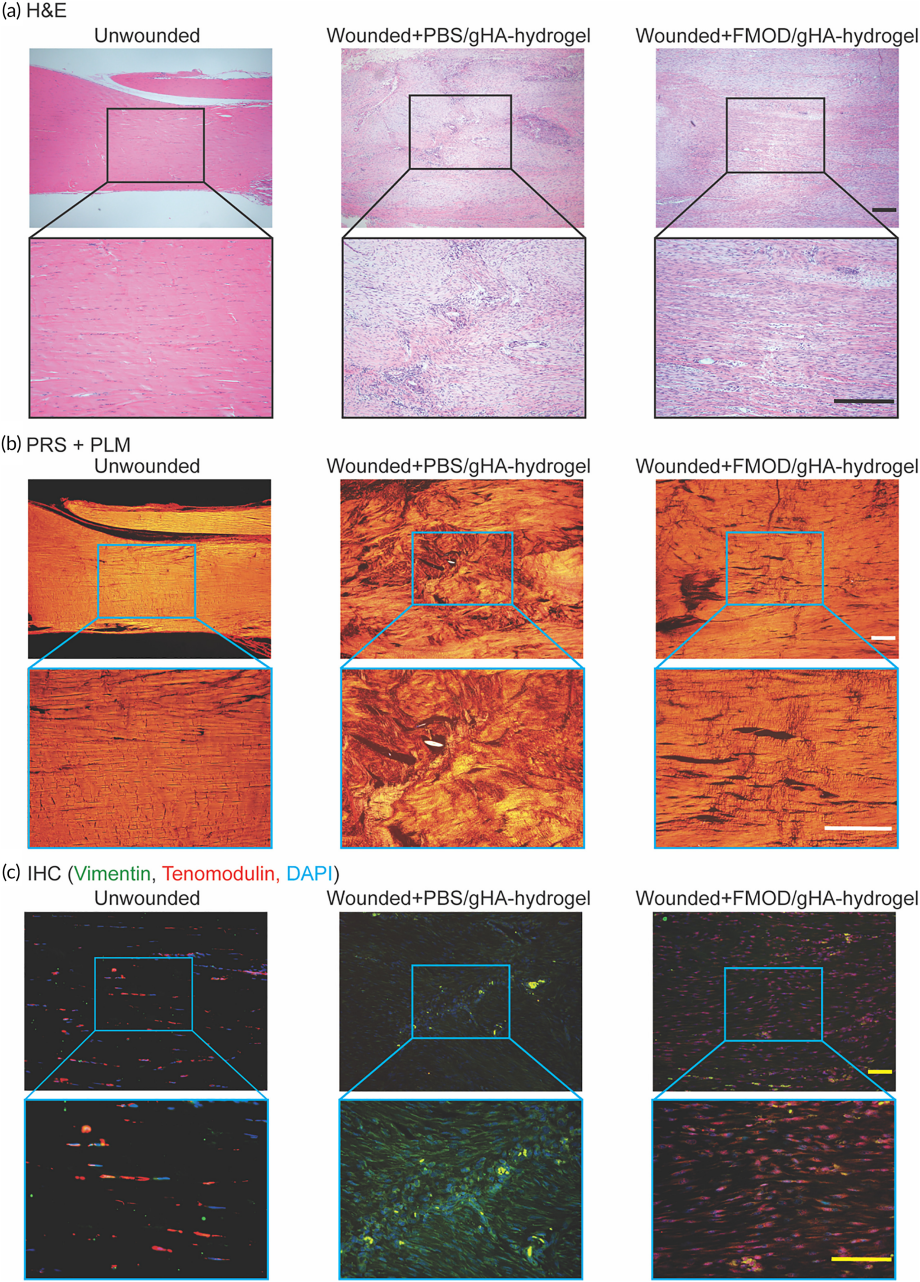
Fibromodulin (FMOD)/gHA‐hydrogel reduced scar formation in adult rat tendon wounds on 21 days post‐injury. Representative H&E staining (a) and picrosirius red (PSR) staining coped polarized light microscopy (PLM) (b) photographs showed FMOD/gHA‐hydrogel‐treated wound have more organized collagen fibrils compared to PBS‐reconstituted gHA‐hydrogel control. Moreover, immunofluorescence staining presented more tenocytes in FMOD/gHA‐hydrogel‐treated tendons (c). Scale bar = 200 μm (a, b) or 25 μm (c).

Damaged or torn tendons are often filled with scar tissue accompanied by abundant and haphazardly arranged collagen fibers,^72^ which was observed in the phosphate‐buffered saline (PBS)‐reconstituted gHA‐hydrogel (Control)‐treated rat tendon at 21 days post‐injury, as displayed by the hematoxylin and eosin (H&E) staining and validated by Picrosirius red (PSR) staining coped polarized light microscopy (PLM) (Figure 5a,b). Thus, the benefit of HA for tendon healing may be negligible. On the other hand, FMOD (constituted in PBS)/gHA‐hydrogel significantly reduced the tendon scarring and led to an organized collagen architecture (Figure 5a,b). Importantly, like the unwounded tendons,^70^ FMOD/gHA‐hydrogel‐treated wounded tendons had longitudinally aligned collagen fibers and exhibited periodic banding under PLM (Figure 5b), suggesting a better reconstruction and load‐bearing capacity recovery.

Next, the collagen architecture was captured by confocal laser scanning microscopy (CLSM) with PSR staining (Figure S5A) and further quantified by a topological method, which is more sensitive than traditional approaches (such as PLM, X‐ray diffraction, laser scattering, and Fourier transform analysis) and has been successfully applied in assessing dermal collagen architectures previously.^29^, ^30^, ^32^, ^73^ The topological feature fractal dimension (*F*_*D*_) provides a scale‐independent measure of how completely an object fills space, quantifying an object by shape, regularity, and lack of smoothness. Compared with the Control group, a significantly higher *F*_*D*_ value was detected on the collagen architecture of FMOD/gHA‐hydrogel‐treated wounded tendons (Figure S5B) indicating a uniform distribution of collagen fibers.^74^ Meanwhile, the topological feature lacunarity (*L*) presents an analysis of density, packing, or dispersion through scales.^74^ A lower *L* value displayed by the collagen architecture of FMOD/gHA‐hydrogel‐treated wounded tendons (Figure S5C), illustrating a finer texture. In contrast, the Control group had a higher *L* value (Figure S5C) that describes a more spatially unorganized collagen architecture.^75^

Liu et al. indicate that fibroblast and tenocyte‐engineered tendons are similar with each other in their gross view, histology, and tensile strength in a porcine model.^76^ Thus, it is important to confirm if mature tenocytes were the predominant cell type recruited by FMOD administration in the wounded tendon site. To achieve this goal, tenomodulin (Tnmd), whose expression is considered to be specific for mature tenocytes (Figure S6),^72^, ^77^, ^78^, ^79^, ^80^ was co‐stained with a general fibroblast marker vimentin.^81^ Minimal Tnmd^+^ cells were found in healing tendons treated with PBS‐reconstituted gHA‐hydrogel (Figure 5c), again indicating HA alone hardly assists functional tendon healing. On the contrary, most cells in the FMOD/gHA‐hydrogel group stained positively with Tnmd (Figure 5c), verifying the pro‐motility potency of FMOD on tenocytes seen in cell culture (Figure 1). In conclusion, a better histological outcome in tendon healing was promoted by FMOD/gHA‐hydrogel application supporting the hypothesis that not only induces an aligned fine extracellular collagen deposition. FMOD also promotes more tenocytes migrating into the wounded site to accelerate tendon healing.

### 2.5 FMOD/gHA‐hydrogel lead to the mechanical property recovery in the wounded tendon

**FIGURE 6:**
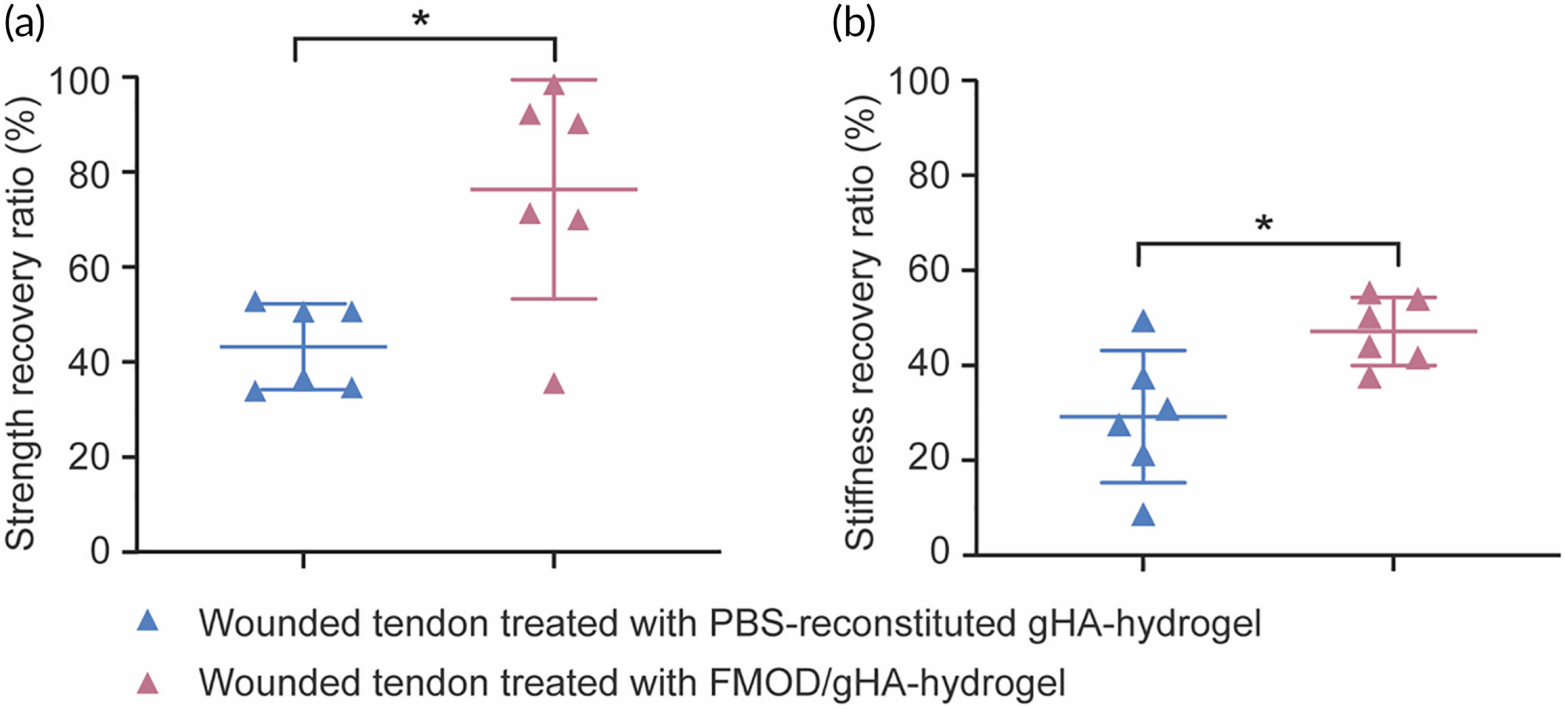
Fibromodulin (FMOD)/gHA‐hydrogel significantly improved the outcome of rat Achilles tendon healing mechanically. A significantly higher strength recovery ratio (a) and stiffness recovery ratio (b) was found in the FMOD/gHA‐hydrogel‐treated tendons in comparison with the control group on 21 days post‐injury. Data are shown as mean ± SD, *n* = 6. Mann–Whitney *U* tests were used for statistical analyses. *p < 0.05.

FMOD/gHA‐hydrogel application resulted in the organized collagen fibrils parallel to the tendon axis (Figure 5), which conferred the tendon's mechanical properties and allowed tendons to resist tensile stress,^82^ and significantly accelerate the mechanical property recovery of the healing tendon (Figure 6). Like the unwounded tendons in which the tissue broke in the middle of the test tendon area, the failures occurred in the middle of the wounded tendons in both the Control and FMOD/gHA‐hydrogel groups. However, compared to the Control group, FMOD/gHA‐hydrogel led to a significant increase in the recovery ratio regarding breaking strength (FMOD/gHA‐hydrogel induces a 76.36% breaking strength recovery ratio, which is 1.76‐times the Control group's recovery ratio [43.24%]; Figure 6a) and stiffness (FMOD/gHA‐hydrogel induces a 47.12% stiffness recovery ratio, which is 1.61‐times the Control group's recovery ratio [29.2%]; Figure 6b).

### 2.6 FMOD/gHA‐hydrogel accelerated functional rehabilitation of Achilles tendon‐injured rats

No doubt, histological and mechanical improvements are important outcomes for tendon wound healing therapies, while activity recovery is more desired by patients suffering from tendon injuries. The rats' walking activity mainly relies on posterior calf muscle contractions to move their toes while the Achilles tendon connects the calf muscles to the calcaneus. Therefore, the gait test is a powerful tool for functionally assessing the recovery of wounded Achilles tendons in our present study.^83^

**FIGURE 7:**
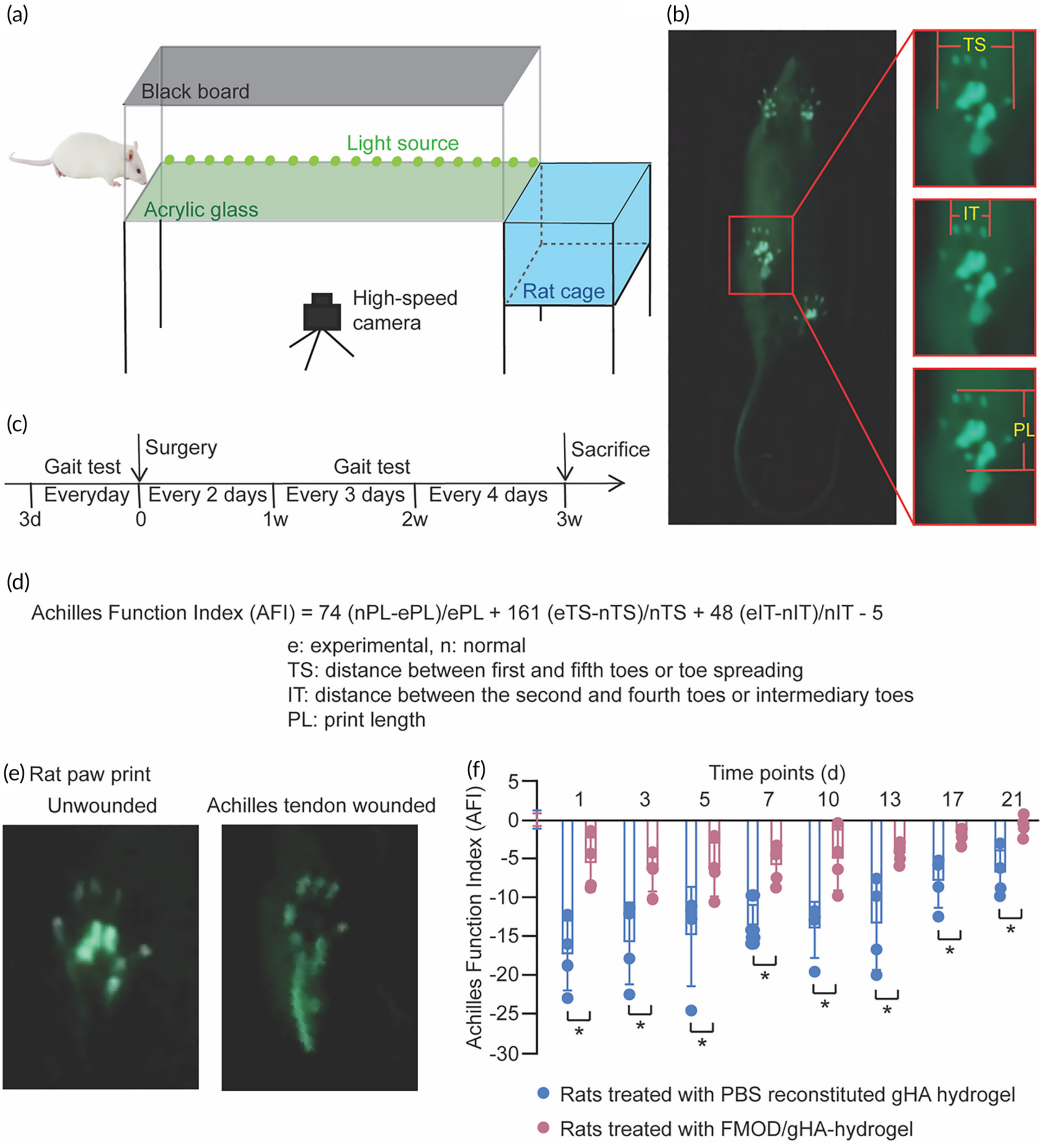
Gait test illustrated the functional recovery of the tendon wounded rats. A gait test apparatus was made according to Cesar S Mendes et al. (a), and the rat's gaits were recorded by videos (b) according to scheduled time points (c). Three parameters, PL (pawprint length), TS (the distance between the first and fifth toes or toe spreading), and IT (the distance between the second and fourth toes or intermediary toes), were measured for AFI calculation (d). Pawprints of unwounded and wounded rats were significantly different (e). AFI were normalized to untreated rats at day 0 (f). Data were shown as mean ± SD, *n* = 4. Mann–Whitney *U* tests were used for statistical analyses. *p < 0.05.

In the present study, a gait test apparatus was built according to the previous description (Figure 7a)^84^ and the rats' movement was documented by video (Figure 7b and Video S2). Furthermore, to better assess the functional rehabilitation of injured rats, gait tests were conducted repeatedly during the 21‐day recovery period (Figure 7c) and quantified by Achilles Functional Index (AFI; Figure 7d)—a precise, reliable, and noninvasive measurement for Achilles tendon functionality.^85^ In accordance with previous studies,^83^ immediately after the tendon injury, Achilles tendon‐wounded rats have a comparably longer and narrower pawprint (Video S3) than healthy rats (Video S2, Figure 7e) associated with a crippled walking posture indicating reduced pressure and maximal area of paw contact led by the impaired ability of toe spreading. Excitingly, compared to the Control group, FMOD/gHA‐hydrogel‐treated animals exhibited significantly higher AFI throughout the entire recovery period (Figure 7f) and demonstrating better tendon function.^85^ In particular, the movement function of the injured Achilles tendons was completely rehabilitated on day 21 post‐injury in FMOD/gHA‐hydrogel group while animals treated with PBS‐reconstituted gHA‐hydrogel still suffered from impaired motility (Figure 7f).

## 3 DISCUSSION

Tendons are a tough band of fibrous connective tissue that attach muscles to bones in the human body. The forces applied to a tendon can be more than five times that of bodyweight. In addition, tendon injuries have become common clinical complications due to overuse of age‐related degeneration.^13^ In some circumstances, such as steroid injection, gout, and hyperparathyroidism, tendons can snap or rupture potentially resulting in excruciating pain and permanent disability. Damaged tendons heal slowly and rarely retain a healthy tendon's structural integrity and mechanical strength. Current medical and surgical treatments are disappointedly nowhere close to regaining full tendon function.^6^, ^13^ Strikingly, corticosteroid injection, a commonly used form of therapy for tendon injury, is also a known intriguing factor for tendon rupture.^6^, ^86^, ^87^ Besides, a diversity of growth factors involved in different phases of the healing process (such as morphogenetic proteins [BMPs], fibroblast growth factor [FGF]2, insulin‐like growth factor [IGF]1, platelet‐derived growth factor [PDGF], transforming growth factor [TGF]βs, and vascular endothelial growth factor [VEGF]), and collagenases and gelatinases (such as MMPs, ADAMs, ADAMTSs, and their endogenous antagonists TIMPs) have also been investigated to enhance tendon repair. However, no major clinical improvement has been achieved despite the immense progress in deciphering the regulatory network governing tendon healing.^6^, ^13^ Meanwhile, stem cell therapies are also applied especially when the tendon stem/progenitor cells (TSPCs) have been identified.^88^, ^89^, ^90^, ^91^, ^92^, ^93^, ^94^, ^95^ However, using TSPCs for tendon repair represents a “*rob Peter to pay Paul*” scenario, and the limited availability as well as the invasive and painful harvesting procedure markedly hinder its application. In addition, using other mesenchymal stem cells (MSCs) is also questionable due to their tumor supporting nature (reviewed in Reference [96]).

Largely consisting of collagens and proteoglycans, tendon is a bundle of uniaxially arranged fibers whose mechanical properties depend on its matrix assembly, which is predominantly determined by collagen fibrillogenesis and regulated by proteoglycans.^24^, ^97^ Recently, accumulating evidence indicates that ECM proteoglycans, particularly SLRPs, play vital roles in regulating ECM assembly and cell activities.^30^, ^32^, ^88^, ^98^, ^99^, ^100^, ^101^ For example, deficiency in either type I (decorin [DCN] and biglycan [BGN]) or type II (FMOD and lumican [LUM]) SLRP leads to collagen fibril disorganization and calcification within the tendon.^24^, ^102^, ^103^, ^104^, ^105^ In the present study, we further verified that FMOD's migratory‐enhancing and ECM‐assessable‐optimizing biopotencies that benefit skin wound healing are extended to tendon healing, presenting FMOD as a bioactive molecule that augments tendon rehabilitation.

As an advantage technology, gene therapy is an appealing approach for therapeutic molecule administration, especially in animal studies.^106^ However, in addition to the expensive and complicated manufacturing barriers, the risk of genetic material transfection in human cells, whether viral vectors are used or not, is seriously concerning.^107^, ^108^, ^109^ The safety incredulity of gene therapy is even worsening recently due to the newly discovered polymerase *θ*, a unique DNA polymerase‐helicase fusion protein that can convert RNA segments back into DNA in mammalian cells.^110^, ^111^ Given the fast development of low‐cost protein production,^112^, ^113^ using the protein form of the therapeutic agent, such as in the case of FMOD—a native ECM molecule broadly distributed in connective tissues,^114^, ^115^ may be much safer and more financially efficient for human usage.

In an injured tendon, the motility direction of tenocytes moving into the wounded site is a fundamental process for tendon repair.^116^, ^117^, ^118^ Unlike dermal fibroblasts, whose migration and invasion were both boosted by FMOD,^30^ tenocytes' migration was only slightly enhanced by FMOD indicating a cell‐specific biopotency of FMOD. It is worth noting that FMOD also significantly promoted tenocyte invasion through collagen matrix, supporting the hypothesis that migration and invasion represent different characters of cell motility even though invasion depends on migration.^118^, ^119^, ^120^ For example, the mechano‐growth factor (MGF) promotes tenocyte migration via decreasing the formation of pseudopodia and F‐actin while enhancing tenocyte invasion by upregulating the production of matrix‐degrading proteinases thus enabling cells to penetrate the ECM.^118^, ^119^ Unlike MGF, whose pro‐invasion effects on tenocytes is relied on increasing MMP2 activity,^118^ FMOD only slightly enhanced *MMP2* expression 24‐h post‐treatment indicating a different mechanism of action (MOA) for FMOD to promote tenocyte invasion such as a previously unreported MMP9‐dependent pathway. Noticeably, MMP3 is thought to play a more prevailing role in tendon ECM remodeling and tissue repair^46^, ^54^ and holds a regulatory function on other MMPs.^46^ Whether FMOD‐induced MMP9 increase relies on MMP3 upregulation is an interesting question to be answered in the near future while uncovering the other signaling mediators of FMOD‐responsive MMP3/9 boost.

Migrating into the wounded site, tenocytes act as a mechanosensory that convert mechanical stimuli into biochemical signals to regulate ECM metabolism and thus determine the tendon's mechanical properties.^82^, ^121^, ^122^, ^123^ Therefore, by enhancing tenocytes' motility, FMOD not only accelerates tendon wound healing but also facilitates tendon collagen fibrils arrangement in alignment with the mechanical force direction. The inverse correlation between MMPs (MMP3 and MMP9) and collagens (*Col1α1* and *Col3α1*) led by FMOD indicates that FMOD accelerates tendon healing via facilitating tenocytes to the wound area promptly and reducing fibrosis/scar formation by prohibiting excessive collagen synthesis and deposition, which is similar to the mechanism that FMOD benefits in skin wound healing.^30^, ^31^ When binding to the growing fibril surface at the early stage of fibril formation, FMOD itself stabilizes small‐diameter fibrils intermediates, prevents premature cross‐linking and small diameter collagen fibrils formation which enhances collagen interconnectivity and fibril stability.^25^, ^27^, ^124^, ^125^ FMOD can benefit tendon healing in two folds by decreasing type I collagen that increases scar formation^30^ and type III collagen that induces thinner collagen architecture leading to tensile strength reduction and increasing the rupture risk of the tendon.^110^ FMOD may also enhance the covalent cross‐linking of collagen and elastin in tendon ECM to accelerate stable collagen fibrils formation^27^ by expedited tenocyte LOX upregulation as LOX is a determining factor for tendon strength via mediating the covalent intermolecular cross‐linking.^25^, ^126^ Taken together, FMOD exhibits promising potential as a pro‐healing agent for tendon injuries at the cellular and molecular levels.

HA is a linear polysaccharide composed of repeating disaccharide units of _D_‐glucuronic acid and N‐acetyl‐_D_‐glucosamine linked by β‐1‐3 and β‐1‐4 glycosidic bonds with several unique functions. For instance, HA facilitates ECM water retention and nutrient diffusion, supports cell proliferation and migration, supports growth factor, drug release, and minimizes mechanical irritation to surrounding tissues.^127^ As a primary ECM component,^128^ HA has intrigued scientists since it was discovered because of the breadth of biological roles it plays despite its chemical simplicity. In particular, HA has been used to treat various musculoskeletal conditions, such as osteoarthritis, rheumatoid arthritis, and tendon injury.^39^, ^127^, ^129^ Together, its biocompatibility, biodegradability, and lack of immune response when implanted into the human body make HA a medically suitable material for releasing FMOD in vivo to improve tendon healing. However, previous studies suggest that native HA is not usable pharmacologically and must first be cross‐linked to provide stability and improve its functionality.^130^, ^131^ Besides, a bulk HA formula is not an optional choice for tendon healing as a minimally invasive procedure requires therapeutics for it to be administered into arbitrarily shaped defects.^132^, ^133^ Thus, a granular HA hydrogel was generated in this study for effective FMOD delivery into the wounded tendon. By fabricating and packing HMPs, we introduced the injectability to the engineered gHA‐hydrogel, which holds proper viscosity to potentially stick on the tissue surface and fill the arbitrarily shaped tendon defects. In addition, the gHA‐hydrogel has a great water‐absorption capability and permits a burst‐to‐sustained two‐phase release of FMOD, resulting in a prompt FMOD delivery followed by a constant dose maintaining period. With all these favorable characteristics of a delivery vehicle, FMOD/gHA‐hydrogel significantly enhances tendon healing, not only histologically and mechanically but also functionally, as evidenced in a rat model with a fully ruptured Achilles tendon. Particularly, after a 21‐day recovery period, the gait performance of rats whose injured tendon was treated with FMOD/gHA‐hydrogel returned to the same level as unwounded animals. Together, this engineered FMOD/gHA‐hydrogel presents the outstanding potential for a new therapeutic candidate for tendon healing management, while the gHA‐hydrogel may have a broader application as a delivery vehicle itself.

As a pre‐clinical proof‐of‐concept investigation, our present study has its limitations. From a basic research standpoint, in addition to tenocytes, FMOD is known to be essential for organizing TSPC niche and modulating TSPCs.^88^ Whether it can contribute to wounded tendon reconstruction is a valuable question to be answered in the future. Besides, FMOD exhibits immunoregulatory potential in skin wound healing.^134^ Understanding if FMOD also regulates the immune response during tendon recovery, particularly at the early healing period, will help to fully reveal how FMOD benefits tendon healing. From a translational standpoint, advanced technologies, such as computational analysis and design and 3D bioprinting,^62^, ^135^ could be adopted to further optimize the FMOD/gHA‐hydrogel manufacture. Moreover, delicate hydrogel designation, such as the pore size, packing density, surface modification, and FMOD dosage should be further optimized and evaluated in different time points of the tendon healing process. Given the high homology between human and rodent FMOD proteins (positives: 100.0%, identity: 91.2%), conventional methods, such as immunostaining, failed to track the release of exogenous FMOD releasing. If necessary, isotope‐labeled FMOD could be used for future preclinical safety assessments and efficacy optimization in the future. From a clinical standpoint, the efficacy of the FMOD/gHA‐hydrogel should also be evaluated in other tendon injuries such as rotator cuff tendon rupture, quadriceps tendon rupture, and biceps tendon rupture. Another area of exploration would be if other SLRPs can aid in tendon rehabilitation. No doubt, a worldwide collaboration among biomolecular scientists, material experts, and surgeons is necessary to make the bench‐to‐bedside transition of this promising therapy.

## 4 MATERIALS AND METHODS

### 4.1 Tenocyte isolation and maintenance

All animal surgery‐related experiments were performed under institutionally approved protocols provided by Chancellor's Animal Research Committee (ARC) at UCLA (protocol number: 2016‐049).

After euthanasia, the Achilles tendons of 3‐months old female Sprague–Dawley rat hind limbs were dissected. Only the middle 1 of 2 tendon proper was collected while the peritendinous connective tissue was removed completely.^133^ The collected tendon tissues were minced into 1 mm^3^ pieces in sterile PBS. After digesting in 2 mg ml^−1^ collagenase type I (Thermo Fisher Scientific) for 4 h at 37°C, transferring the digested tendon tissues into a 10‐cm culture dish (Thermo Fisher Scientific) with Tenocyte Culture Medium (ZenBio, Inc, Durham, NC, USA), 10% fetal bovine serum (FBS; Thermo Fisher Scientific), penicillin/streptomycin (1% v/v; Thermo Fisher Scientific), and amphotericin B (1% v/v; Thermo Fisher Scientific) for 1 week. Then the tendon tissue and the isolated cells were removed into a new 10‐cm culture dish in a 37°C incubator with 5% CO_2_ after digesting and collecting with 0.5% trypsin (Thermo Fisher Scientific). Tenocytes at passage 3 were used for all in vitro tests.^118^, ^132^ All tenocytes used in this study were mycoplasma negative which was validated by good laboratory practice (GLP)‐Compliant Validation of MycoDetective™ ‐Mycoplasma Detection Using Real‐time qPCR (Laragen Inc., CA, USA).

### 4.2 Injectable hydrogel manufacture

The hydrogel was prepared by cross‐linking HA polymer with DVS to form a 3D network structure (Figure 3). First, 4% HA polymer (Mw, ~1500–1800 kDa; MilliporeSigma) dissolved in 0.2 M NaOH and DVS (purity ≥ 96%; MilliporeSigma) were mixed at a ratio of 4:1 (w/w) with magnetic stirring for 0.5 h to cross‐link (Figure 3a). Then, the mixture was neutralized with an equivalent amount of 0.2 M HCl and dialyzed against pure water for 72 h (Figure 3b). To obtain the injectable HMPs that can pass through the syringe needles, the hydrogel after dialysis was strained through 100‐mesh Standard Stainless‐Steel Sieve (Thermo Fisher Scientific) (Figure 3c). The HA HMPs hydrogel was sterilized by immersing into the 75% ethanol solution for 2 h. After the ethanol was replaced by sterilized distilled water, HA HMPs were lyophilized to form granular hydrogel^60^ and then stored at 4°C (Figure 3d). Prior to the administration, the injectable, gHA‐hydrogel was reconstituted with 6 mg ml^−1^ FMOD solution (Figure 3e).

### 4.3 Adult rat tendon wounds model

Female Sprague–Dawley rats (with age 120 days, weighting about 300 g) were randomly and equally assigned into two groups: (1) Control group: PBS‐reconstituted gHA‐hydrogel, and (2) FMOD group: FMOD/gHA‐hydrogel containing 12 mg ml^−1^ FMOD. After anesthetizing, a longitudinal skin incision was made over the sterilized right hind limb to expose the Achilles tendon **(**Figure S4A), while the left hind limb was kept unwounded for gait performance assessment and used as an intra‐animal control for mechanical testing.^85^ After transecting the right Achilles tendon in the middle (Figure S4B), a 5 μl of reconstituted, gHA‐hydrogel was placed on each wound edge (5 μl × 2 edges = 10 μl total/wound). The tendon was then sutured with 5–0 Nylon by using modified 2‐strand Kirchmayr–Kessler technique (Figure S4C–D),^136^ and the skin incision was closed in layers with interrupted suture (Figure S4E). Cutaneous sutures were removed 1‐week post‐injury when stable wound closure established.^31^, ^99^, ^134^ Measurements were carried out by the investigators who were blinded to the treatment group assignment. Rats were sacrificed 21 days post‐operation.

### 4.4 Histological and immunofluorescence staining

The harvested Achilles tendons were fixed in 4% paraformaldehyde (PFA) at room temperature for 24 h. To ensure a more precise quantification, wounds were bisected centrally and longitudinally. After dehydration, samples were paraffin‐embedded and sectioned as 5‐μm for the H&E staining, and immunofluorescence staining,^137^ while 10‐μm slides were prepared for PSR staining.^30^ To keep the region of interest (ROI) with a consistent location in the tendon, an unwounded tendon from the left leg was used as a control substance. Particularly, all the samples were placed in the same direction on the slides for histological assessment: the end connected to the calcaneus originated right in the photos while the end connected to the calf muscle originated left (Figure S4G,H). The longitudinal middle of each scar was documented for further assessments. The primary antibodies used for Immunohistochemistry staining are listed in Table S2.

### 4.5 Confocal laser scanning microscopy)

Following PSR staining, the collagen architecture of the tendon tissue was captured on a Leica TCS‐SP5 AOBS confocal microscope (Leica Microsystems, Wetzlar, Germany) equipped with the software Leica Application Suite Advanced Fluorescence (LAS AF; Leica Microsystems).^31^, ^32^, ^73^ Images were then assessed with Image J for *F*_*D*_ and *L* analyses to quantify the topological structure of the collagen network.^73^

### 4.6 Biomechanical analysis

Rats were sacrificed at 21 days post‐injury. Both wounded and unwounded hind Achilles tendons together with calcaneus and 1 cm proximal muscle were harvested and tested within 2 h from the harvest time point. Adherent surrounding tissues and other muscles were removed completely. Ultimate tensile strength was tested using an Instron 5565 Universal Testing Machine (Instron, High Wycombe, UK). The tendon specimen was fixed by two clamps of Instron. One clamp was placed at the musculotendon junction and the other was placed at the attachment of Achilles tendon to the calcaneus. The distance between the clamps is 10 mm. Pneumatic grips and silicon carbide waterproof papers were used as padding to avoid specimen slippage.

(1) $$\text{Strength recovery ratio}=S_{p}/S_{0}\times 100\%$$

Here, the *S*_p_ and *S*_0_ represent the breaking strength at 21st days postoperation and preoperation, respectively.

A linear portion of the elastic phase of the curve was marked and tendon stiffness (N/mm) was calculated from the slope of the force‐displacement curve.^138^, ^139^

(2) Stiffness recovery ratio=F/D×100%

Here, F represents the force applied to the Achilles tendon in the tendon's elastic phase and D represents the displacement the tendon experiences when the force is applied.

### 4.7 Gait performance evaluation

The gait test apparatus was made according to Mendes et al. (Figure 7a).^84^ Rats were placed in a confined acrylic glass gait walkway build with transparent floor and sides (100 cm × 20 cm) together with LED lights attached at the edge of the acrylic glass floor. Rat footprints disrupt the internal reflection of the LED light propagating within the acrylic glass and are recorded by a high‐speed video camera (Figure 7b). Rats were trained to walk through the walkway in a consistent velocity while the animals failing the walk training were excluded. Rat footprints were measured to evaluate the functional recovery of the injured Achilles tendon, while the gait performance of the animals were recorded every day since 3 days prior to the surgery to settle the baseline (Figure 7c).^85^ A full‐length walk with usual and consistent velocity, without any interruption or hesitation, will be considered a successful walk (Video S2) and three successful walks for each rat were recorded for each test. Only rats that performed three successful continuous gait tests were used for further gait parameter evaluation. Footprint length (PL), distance between the first and fifth toes or toe spreading (TS) and distance between the second and fourth toes or toe spreading (IT) were measured (Figure 7b) as described by de Medinaceli et al. previously.^140^ Only footprints in the middle of the walkway were used for analysis. AFI described by Murrell et al. (Figure 7d)^85^ was assessed and normalized to the pre‐surgery level:

(3) $$\text{Achilles Functional Index}\;\left(\mathrm{AFI}\right)=74\times \left(\mathrm{PLF}\right)+161\times \left(\mathrm{TSF}\right)+48\times \left(\mathrm{ITF}\right)-5$$

Here,

(4) $$\mathrm{de}\;{\text{Medinaceli}}^{'}s\;\text{footprint length factor}\;\left(\mathrm{PLF}\right)=\left(n\mathrm{PL}-e\mathrm{PL}\right)/\mathrm{ePL}$$

(5) $$\mathrm{Toe}-\text{spread factor}\;\left(\mathrm{TSF}\right)=\left(e\mathrm{TS}-n\mathrm{TS}\right)/n\mathrm{TS}$$

(6) $$\text{Intermediary}\;\mathrm{toe}-\text{spread factor}\;\left(\mathrm{ITF}\right)=\left(e\mathrm{IT}-n\mathrm{IT}\right)/n\mathrm{IT}$$

In which, *n*: unwounded limb; *e*: wounded limb.

### 4.8 Statistical analysis

All statistical analyses were conducted in consultation with the UCLA Department of Medicine Statistics Core. Power analysis by GPower (version 3.1.9.4, Franz Faul, Universitat Kiel, Germany) was used to predict the sample used in the present study to ensure α = 0.05 and power = 0.8. As a proof‐of‐concept study, the power analysis was conducted with a Cohen's *d* = 2.1 (>2, the threshold for a “‘Huge” effect) to secure the clinical significance of the presentt study. Parametric data were compared by one‐way ANOVA and two‐sample *t*‐tests (one‐tailed) using GraphPad Prism 9.0.0 (GraphPad Software, LLC, CA, USA), while one‐tailed Mann–Whitney *U* test and Kruskal–Wallis ANOVA tests were used for non‐parametric data. For all data presented in this article, *p* < 0.05 (*) was considered a suggestive difference, while *p* < 0.005 (**) was recognized as a statistical significance.^141^ No data was excluded. The relative statistical analysis information is also presented in the respective figure legends.

## 5 CONCLUSIONS

Our present study proved the hypothesis that FMOD's migratory‐enhancing and ECM‐assembly‐optimizing bioactivities in skin wounds are duplicated in the tendon healing process. An injectable gHA‐hydrogel was engineered to effectively deliver this bio‐potent agent in the wounded tendon through a minimally invasive procedure desired by both patients and healthcare providers. The gHA‐hydrogel has an excellent water‐absorption capability to keep the administrated FMOD and has enough viscosity to stick to tissue surfaces and fill the arbitrarily shaped tendon defects. Moreover, the reconstituted FMOD/gHA‐hydrogel displays a burst‐to‐sustained two‐phase release of FMOD. Thus, a single administration of the FMOD/gHA‐hydrogel is sufficient and permits a prompt delivery of FMOD followed by a constant dose‐maintaining period. By using a rat's Achilles tendon injury model, we demonstrate that our FMOD/gHA‐hydrogel significantly augmented tendon healing, histologically, mechanically, and functionally. FMOD is a native ECM component that is broadly distributed in the connective tissue, and multiple DVS cross‐linked HA hydrogels have been approved for human usage; therefore, FMOD/gHA‐hydrogel is a promising tendon‐healing therapeutic agent in the clinical setting.

## AUTHOR CONTRIBUTIONS

**Zhong Zheng:** Conceptualization (lead); data curation (lead); formal analysis (lead); funding acquisition (lead); methodology (equal); resources (equal); writing – review and editing (lead). **Xue Xu:** Data curation (lead); formal analysis (lead); investigation (lead); methodology (equal); validation (lead); writing – original draft (lead). **Yulong Zhang:** Data curation (lead); investigation (lead); methodology (lead); validation (lead). **Pin Ha:** Data curation (equal); investigation (equal); methodology (equal); validation (equal). **Yao Chen:** Investigation (equal). **Chenshuang Li:** Investigation (equal). **Emily Yen:** Writing – review and editing (equal). **Yuxing Bai:** Project administration (supporting). **Renji Chen:** Project administration (supporting). **Benjamin M. Wu:** Methodology (supporting). **Andrew Da Lio:** Methodology (supporting). **Kang Ting:** Conceptualization (lead); Methodology (equal); Resources (lead); Writing – Review & Editing (equal); Supervision (lead). **Chia Soo:** Conceptualization (lead); data curation (equal); formal analysis (lead); funding acquisition (lead); methodology (equal); resources (lead); writing – review and editing (equal).

## FUNDING INFORMATION

This study was financially supported by NIH Center for Dental, Oral, and Craniofacial Tissue and Organ Regeneration (C‐DOCTOR) Interdisciplinary Translational Project (ITP) Team Award (U24DE026914) and Plastic Surgery Foundation, Translational Research Grant (#571906). Confocal laser scanning microscopy was performed at the Advanced Light Microscopy/Spectroscopy Laboratory and the Leica Microsystems Center of Excellence at the California NanoSystems Institute at UCLA with funding support from NIH Shared Instrumentation Grant S10OD025017 and NSF Major Research Instrumentation grant CHE‐0722519.

## CONFLICT OF INTEREST

Drs. Kang Ting, Chia Soo, and Zhong Zheng are the inventors on FMOD‐related patents assigned to UCLA. Drs. Kang Ting, Chia Soo, and Zhong Zheng are also founders and officers of Scarless Laboratories, Inc., which sublicenses FMOD‐related patents from the UC Regents, who also hold equity in the company.

### PEER REVIEW

The peer review history for this article is available at https://publons.com/publon/10.1002/btm2.10355.

## Supporting information


**Appendix S1** Supporting Information

**Video S1** 

**Video S2** 

**Video S3** 

## Data Availability

Datasets generated and/or analyzed during this study are available from the corresponding author on reasonable request.

## References

[1] Voleti PB , Buckley MR , Soslowsky LJ . Tendon healing: repair and regeneration. Annu Rev Biomed Eng. 2012;14:47‐71. 22809137 10.1146/annurev-bioeng-071811-150122

[2] Docheva D , Muller SA , Majewski M , Evans CH . Biologics for tendon repair. Adv Drug Deliv Rev. 2015;84:222‐239. 25446135 10.1016/j.addr.2014.11.015 PMC4519231

[3] September AV , Schwellnus MP , Collins M . Tendon and ligament injuries: the genetic component. Br J Sports Med. 2007;41:241‐246. discussion 246. 17261551 10.1136/bjsm.2006.033035 PMC2658952

[4] Yan Z , Yin H , Nerlich M , Pfeifer CG , Docheva D . Boosting tendon repair: interplay of cells, growth factors and scaffold‐free and gel‐based carriers. J Exp Orthop. 2018;5:1. 29330711 10.1186/s40634-017-0117-1 PMC5768579

[5] Lemme NJ , Li NY , DeFroda SF , Kleiner J , Owens BD . Epidemiology of achilles tendon ruptures in the United States: athletic and nonathletic injuries from 2012 to 2016. Orthop J Sports Med. 2018;6: 2325967118808238. 10.1177/2325967118808238 PMC6259075 30505872

[6] Lipman K , Wang C , Ting K , Soo C , Zheng Z . Tendinopathy: injury, repair, and current exploration. Drug des Devel Ther. 2018;12:591‐603. 10.2147/DDDT.S154660 PMC5865563 29593382

[7] Kaux JF , Forthomme B , Goff CL , Crielaard JM , Croisier JL . Current opinions on tendinopathy. J Sports Sci Med. 2011;10:238‐253. 24149868 PMC3761855

[8] Scott A , Ashe MC . Common tendinopathies in the upper and lower extremities. Curr Sports Med Rep. 2006;5:233‐241. 16934204 10.1097/01.csmr.0000306421.85919.9c

[9] Maffulli N , Longo UG , Loppini M , Denaro V . Current treatment options for tendinopathy. Expert Opin Pharmacother. 2010;11:2177‐2186. 20569088 10.1517/14656566.2010.495715

[10] Ramos D , Peach MS , Mazzocca AD , Yu X , Kumbar SG . Tendon tissue engineering. In: Nukavarapu SP , Freeman JW , Laurencin CT , eds. Regenerative Engineering of Musculoskeletal Tissues and Interfaces. Elsevier; 2015.

[11] Pennisi E . Tending tender tendons. Science. 2002;295:1011. 11834816 10.1126/science.295.5557.1011

[12] Abbah SA , Spanoudes K , O'Brien T , Pandit A , Zeugolis DI . Assessment of stem cell carriers for tendon tissue engineering in pre‐clinical models. Stem Cell Res Ther. 2014;5:38. 25157898 10.1186/scrt426 PMC4056691

[13] Wu F , Nerlich M , Docheva D . Tendon injuries: basic science and new repair proposals. EFORT Open Rev. 2017;2:332‐342. 28828182 10.1302/2058-5241.2.160075 PMC5549180

[14] Ackermann PW . Tendinopathy I: understanding epidemiology, pathology, healing, and treatment. In: Gomes ME , Reis RL , Rodrigues MT , eds. Tendon regeneration. Elsevier; 2015:113‐147.

[15] Holm C , Kjaer M , Eliasson P . Achilles tendon rupture ‐ treatment and complications: a systematic review. Scand J Med Sci Sports. 2015;25:e1‐e10. 24650079 10.1111/sms.12209

[16] Lim WL , Liau LL , Ng MH , Chowdhury SR , Law JX . Current progress in tendon and ligament tissue engineering. Tissue Eng Regen Med. 2019;16:549‐571. 31824819 10.1007/s13770-019-00196-w PMC6879704

[17] Lui PP , Rui YF , Ni M , Chan KM . Tenogenic differentiation of stem cells for tendon repair‐what is the current evidence? J Tissue Eng Regen Med. 2011;5:e144‐e163. 21548133 10.1002/term.424

[18] Thakkar RS , Thakkar SC , Srikumaran U , McFarland EG , Fayad LM . Complications of rotator cuff surgery‐the role of post‐operative imaging in patient care. Br J Radiol. 2014;87:20130630. 24734935 10.1259/bjr.20130630 PMC4075575

[19] Chen CH , Cao Y , Wu YF , Bais AJ , Gao JS , Tang JB . Tendon healing in vivo: gene expression and production of multiple growth factors in early tendon healing period. J Hand Surg Am. 2008;33:1834‐1842. 19084187 10.1016/j.jhsa.2008.07.003

[20] Molloy T , Wang Y , Murrell G . The roles of growth factors in tendon and ligament healing. Sports Med. 2003;33:381‐394. 12696985 10.2165/00007256-200333050-00004

[21] Yang G , Rothrauff BB , Tuan RS . Tendon and ligament regeneration and repair: clinical relevance and developmental paradigm. Birth Defects Res C Embryo Today. 2013;99:203‐222. 24078497 10.1002/bdrc.21041 PMC4041869

[22] de Aro AA , de Campos VB , Pimentel ER . Biochemical and anisotropical properties of tendons. Micron. 2012;43:205‐214. 21890364 10.1016/j.micron.2011.07.015

[23] Magnusson SP , Hansen P , Kjaer M . Tendon properties in relation to muscular activity and physical training. Scand J Med Sci Sports. 2003;13:211‐223. 12859603 10.1034/j.1600-0838.2003.00308.x

[24] Ezura Y , Chakravarti S , Oldberg A , Chervoneva I , Birk DE . Differential expression of lumican and fibromodulin regulate collagen fibrillogenesis in developing mouse tendons. J Cell Biol. 2000;151:779‐788. 11076963 10.1083/jcb.151.4.779 PMC2169450

[25] Kalamajski S , Liu C , Tillgren V , et al. Increased c‐telopeptide cross‐linking of tendon type i collagen in fibromodulin‐deficient mice. J Biol Chem. 2014;289:18873‐18879. 24849606 10.1074/jbc.M114.572941 PMC4081928

[26] Xue X , Pin H , Emily Y , Chenshuang L , Zhong Z . Small leucine‐rich proteoglycans in tendon wound healing. Adv Wound Care (New Rochelle). 2022;11:202‐214. 34978952 10.1089/wound.2021.0069

[27] Eyre DR , Weis MA , Wu JJ . Advances in collagen cross‐link analysis. Methods. 2008;45:65‐74. 18442706 10.1016/j.ymeth.2008.01.002 PMC2398701

[28] Jepsen KJ , Wu F , Peragallo JH , et al. A syndrome of joint laxity and impaired tendon integrity in lumican‐ and fibromodulin‐deficient mice. J Biol Chem. 2002;277:35532‐35540. 12089156 10.1074/jbc.M205398200

[29] Jiang W , Ting K , Lee S , et al. Fibromodulin reduces scar size and increases scar tensile strength in normal and excessive‐mechanical‐loading porcine cutaneous wounds. J Cell Mol Med. 2018;22:2510‐2513. 29392829 10.1111/jcmm.13516 PMC5867110

[30] Zheng Z , James AW , Li C , et al. Fibromodulin reduces scar formation in adult cutaneous wounds by eliciting a fetal‐like phenotype. Signal Transduct Target Ther. 2017;2:17050. 29201497 10.1038/sigtrans.2017.50 PMC5661627

[31] Zheng Z , Nguyen C , Zhang X , et al. Delayed wound closure in fibromodulin‐deficient mice is associated with increased tgf‐beta3 signaling. J Invest Dermatol. 2011;131:769‐778. 21191417 10.1038/jid.2010.381 PMC4073663

[32] Zheng Z , Zhang X , Dang C , et al. Fibromodulin is essential for fetal‐type scarless cutaneous wound healing. Am J Pathol. 2016;186:2824‐2832. 27665369 10.1016/j.ajpath.2016.07.023 PMC5222972

[33] Buschmann J , Burgisser GM . Biomechanics of tendons and ligaments. Tissue Reconstruction and Regeneration. Elsevier; 2017.

[34] Denegar CR , Saliba E , Saliba S . Therapeutic Modalities for Musculoskeletal Injuries. 4th ed. Human Kinetics; 2015.

[35] Subramanian A , Schilling TF . Tendon development and musculoskeletal assembly: emerging roles for the extracellular matrix. Development. 2015;142:4191‐4204. 26672092 10.1242/dev.114777 PMC4689213

[36] Ahmed EM . Hydrogel: preparation, characterization, and applications: a review. J Adv Res. 2015;6:105‐121. 25750745 10.1016/j.jare.2013.07.006 PMC4348459

[37] Ibrahim M , El‐Sherbiny MHY . Hydrogel scaffolds for tissue engineering: Progress and challenges. Glob Cardiol Sci Pract. 2013;2013:316‐342. 24689032 10.5339/gcsp.2013.38 PMC3963751

[38] Hunt DR , Jovanovic SA , Wikesjo UM , Wozney JM , Bernard GW . Hyaluronan supports recombinant human bone morphogenetic protein‐2 induced bone reconstruction of advanced alveolar ridge defects in dogs. A pilot study. J Periodontol. 2001;72:651‐658. 11394401 10.1902/jop.2001.72.5.651

[39] Liang JI , Lin PC , Chen MY , Hsieh TH , Chen JJJ , Yeh ML . The effect of tenocyte/hyaluronic acid therapy on the early recovery of healing achilles tendon in rats. J Mater Sci Mater Med. 2014;25:217‐227. 24072390 10.1007/s10856-013-5036-9

[40] Song R , Murphy M , Li C , Ting K , Soo C , Zheng Z . Current development of biodegradable polymeric materials for biomedical applications. Drug des Devel Ther. 2018;12:3117‐3145. 10.2147/DDDT.S165440 PMC6161720 30288019

[41] Choi JR , Yong KW , Choi JY , Cowie AC . Recent advances in photo‐crosslinkable hydrogels for biomedical applications. Biotechniques. 2019;66:40‐53. 30730212 10.2144/btn-2018-0083

[42] Nichols AEC , Best KT , Loiselle AE . The cellular basis of fibrotic tendon healing: challenges and opportunities. Transl Res. 2019;209:156‐168. 30776336 10.1016/j.trsl.2019.02.002 PMC6545261

[43] Samiric T , Ilic MZ , Handley CJ . Characterisation of proteoglycans and their catabolic products in tendon and explant cultures of tendon. Matrix Biol. 2004;23:127‐140. 15246111 10.1016/j.matbio.2004.03.004

[44] Andarawis‐Puri N , Flatow EL , Soslowsky LJ . Tendon basic science: development, repair, regeneration, and healing. J Orthop Res. 2015;33:780‐784. 25764524 10.1002/jor.22869 PMC4427041

[45] Magra M , Maffulli N . Matrix metalloproteases: a role in overuse tendinopathies. Br J Sports Med. 2005;39:789‐791. 16244185 10.1136/bjsm.2005.017855 PMC1725078

[46] Riley GP , Curry V , DeGroot J , et al. Matrix metalloproteinase activities and their relationship with collagen remodelling in tendon pathology. Matrix Biol. 2002;21:185‐195. 11852234 10.1016/s0945-053x(01)00196-2

[47] Sharma P , Maffulli N . Biology of tendon injury: healing, modeling and remodeling. J Musculoskelet Neuronal Interact. 2006;6:181‐190. 16849830

[48] Steffensen B , Hakkinen L , Larjava H . Proteolytic events of wound‐healing–coordinated interactions among matrix metalloproteinases (mmps), integrins, and extracellular matrix molecules. Crit Rev Oral Biol Med. 2001;12:373‐398. 12002821 10.1177/10454411010120050201

[49] de Mello Malheiro OC , Giacomini CT , Justulin LA Jr , Delella FK , Dal‐Pai‐Silva M , Felisbino SL . Calcaneal tendon regions exhibit different mmp‐2 activation after vertical jumping and treadmill running. Anat Rec (Hoboken). 2009;292:1656‐1662. 19685505 10.1002/ar.20953

[50] Oshiro W , Lou J , Xing X , Tu Y , Manske PR . Flexor tendon healing in the rat: a histologic and gene expression study. J Hand Surg Am. 2003;28:814‐823. 14507513 10.1016/s0363-5023(03)00366-6

[51] Oblander SA , Zhou Z , Gálvez BG , et al. Distinctive functions of membrane type 1 matrix‐metalloprotease (mt1‐mmp or mmp‐14) in lung and submandibular gland development are independent of its role in pro‐mmp‐2 activation. Dev Biol. 2005;277:255‐269. 15572153 10.1016/j.ydbio.2004.09.033

[52] Page‐McCaw A , Ewald AJ , Werb Z . Matrix metalloproteinases and the regulation of tissue remodelling. Nat Rev Mol Cell Biol. 2007;8:221‐233. 17318226 10.1038/nrm2125 PMC2760082

[53] Strongin AY , Collier I , Bannikov G , Marmer BL , Grant GA , Goldberg GI . Mechanism of cell surface activation of 72‐kda type iv collagenase. Isolation of the activated form of the membrane metalloprotease. J Biol Chem. 1995;270:5331‐5338. 7890645 10.1074/jbc.270.10.5331

[54] Ireland D , Harrall R , Curry V , et al. Multiple changes in gene expression in chronic human achilles tendinopathy. Matrix Biol. 2001;20:159‐169. 11420148 10.1016/s0945-053x(01)00128-7

[55] Nakamura K , Yokohama S , Yoneda M , et al. High, but not low, molecular weight hyaluronan prevents t‐cell‐mediated liver injury by reducing proinflammatory cytokines in mice. J Gastroenterol. 2004;39:346‐354. 15168246 10.1007/s00535-003-1301-x

[56] Simulescu V , Kalina M , Mondek J , Pekar M . Long‐term degradation study of hyaluronic acid in aqueous solutions without protection against microorganisms. Carbohydr Polym. 2016;137:664‐668. 26686177 10.1016/j.carbpol.2015.10.101

[57] FDA U . Dermal Fillers Approved by the Center for Devices and Radiological Healt. FDAU; 2018 https://www.fda.gov/medical‐devices/cosmetic‐devices/dermal‐fillers‐approved‐center‐devices‐and‐radiological‐health/

[58] Kim JT , Lee DY , Kim EJ , Jang JW , Cho NI . Tissue response to implants of hyaluronic acid hydrogel prepared by microbeads. Tissue Eng Regen Med. 2014;11:32‐38.

[59] Daly AC , Riley L , Segura T , Burdick JA . Hydrogel microparticles for biomedical applications. Nat Rev Mater. 2020;5:20‐43. 34123409 10.1038/s41578-019-0148-6 PMC8191408

[60] Qazi TH , Burdick JA . Granular hydrogels for endogenous tissue repair. Biomaterials and Biosystems. 2021;1:100008. 10.1016/j.bbiosy.2021.100008 PMC9934473 36825161

[61] Mealy JE , Chung JJ , Jeong HH , et al. Injectable granular hydrogels with multifunctional properties for biomedical applications. Adv Mater. 2018;30:e1705912. 29602270 10.1002/adma.201705912

[62] Riley L , Schirmer L , Segura T . Granular hydrogels: emergent properties of jammed hydrogel microparticles and their applications in tissue repair and regeneration. Curr Opin Biotechnol. 2019;60:1‐8. 30481603 10.1016/j.copbio.2018.11.001 PMC6534490

[63] Shaikh HK , Kshirsagar R , Patil SG . Mathematical models for drug release characterization: a review. *World* . J Pharm Pharm Sci. 2015;4:324‐338.

[64] Sjöholm E , Mathiyalagan R , Rajan Prakash D , et al. 3d‐printed veterinary dosage forms‐a comparative study of three semi‐solid extrusion 3d printers. Pharmaceutics. 2020;12:1239. 10.3390/pharmaceutics12121239 PMC7767139 33352700

[65] Dash S , Murthy PN , Nath L , Chowdhury P . Kinetic modeling on drug release from controlled drug delivery systems. Acta pol Pharm. 2010;67:217‐223. 20524422

[66] Domb A , Davidson GWR , Sanders LM . Diffusion of peptides through hydrogel membranes. J Control Release. 1990;14:133‐144.

[67] Hsu HH , Kracht JK , Harder LE , et al. A method for determination and simulation of permeability and diffusion in a 3d tissue model in a membrane insert system for multi‐well plates. J Vis Exp. 2018;23:56412. 10.3791/56412 PMC5931342 29553546

[68] Kopac T , Rucigaj A , Krajnc M . The mutual effect of the crosslinker and biopolymer concentration on the desired hydrogel properties. Int J Biol Macromol. 2020;159:557‐569. 32422268 10.1016/j.ijbiomac.2020.05.088

[69] Macha IJ , Ben‐Nissan B , Vilchevskaya EN , et al. Drug delivery from polymer‐based nanopharmaceuticals‐an experimental study complemented by simulations of selected diffusion processes. Front Bioeng Biotechnol. 2019;7:37. 30906737 10.3389/fbioe.2019.00037 PMC6418005

[70] Freedman BR , Gordon JA , Soslowsky LJ . The achilles tendon: fundamental properties and mechanisms governing healing. Muscles Ligaments Tendons J. 2014;4:245‐255. 25332943 PMC4187594

[71] Jarvinen TA , Kannus P , Maffulli N , Khan KM . Achilles tendon disorders: etiology and epidemiology. Foot Ankle Clin. 2005;10:255‐266. 15922917 10.1016/j.fcl.2005.01.013

[72] Shukunami C , Yoshimoto Y , Takimoto A , Yamashita H , Hiraki Y . Molecular characterization and function of tenomodulin, a marker of tendons and ligaments that integrate musculoskeletal components. Jpn Dent Sci Rev. 2016;52:84‐92. 28408960 10.1016/j.jdsr.2016.04.003 PMC5390337

[73] Khorasani H , Zheng Z , Nguyen C , et al. A quantitative approach to scar analysis. Am J Pathol. 2011;178:621‐628. 21281794 10.1016/j.ajpath.2010.10.019 PMC3070584

[74] Smith TG Jr , Lange GD , Marks WB . Fractal methods and results in cellular morphology–dimensions, lacunarity and multifractals. J Neurosci Methods. 1996;69:123‐136. 8946315 10.1016/S0165-0270(96)00080-5

[75] Ling EJ , Servio P , Kietzig AM . Fractal and lacunarity analyses: quantitative characterization of hierarchical surface topographies. Microsc Microanal. 2016;22:168‐177. 26758776 10.1017/S1431927615015561

[76] Liu W , Chen B , Deng D , Xu F , Cui L , Cao Y . Repair of tendon defect with dermal fibroblast engineered tendon in a porcine model. Tissue Eng. 2006;12:775‐788. 16674291 10.1089/ten.2006.12.775

[77] Shukunami C , Takimoto A , Nishizaki Y , et al. Scleraxis is a transcriptional activator that regulates the expression of tenomodulin, a marker of mature tenocytes and ligamentocytes. Sci Rep. 2018;8:3155. 29453333 10.1038/s41598-018-21194-3 PMC5816641

[78] Shukunami C , Takimoto A , Oro M , Hiraki Y . Scleraxis positively regulates the expression of tenomodulin, a differentiation marker of tenocytes. Dev Biol. 2006;298:234‐247. 16876153 10.1016/j.ydbio.2006.06.036

[79] Docheva D , Hunziker EB , Fassler R , Brandau O . Tenomodulin is necessary for tenocyte proliferation and tendon maturation. Mol Cell Biol. 2005;25:699‐705. 15632070 10.1128/MCB.25.2.699-705.2005 PMC543433

[80] Huang AH , Lu HH , Schweitzer R . Molecular regulation of tendon cell fate during development. J Orthop Res. 2015;33:800‐812. 25664867 10.1002/jor.22834

[81] Chen ZY , Yu XF , Huang JQ , Li DL . The mechanisms of beta‐catenin on keloid fibroblast cells proliferation and apoptosis. Eur Rev Med Pharmacol Sci. 2018;22:888‐895. 29509234 10.26355/eurrev_201802_14366

[82] Kjaer M . Role of extracellular matrix in adaptation of tendon and skeletal muscle to mechanical loading. Physiol Rev. 2004;84:649‐698. 15044685 10.1152/physrev.00031.2003

[83] Messner K , Wei Y , Andersson B , Gillquist J , Rasanen T . Rat model of achilles tendon disorder. A pilot study. Cells Tissues Organs. 1999;165:30‐39. 10460971 10.1159/000016671

[84] Mendes CS , Bartos I , Márka Z , Akay T , Márka S , Mann RS . Quantification of gait parameters in freely walking rodents. BMC Biol. 2015;13:50. 26197889 10.1186/s12915-015-0154-0 PMC4511453

[85] Murrell GA , Lilly EG , Davies H , Best TM , Goldner RD , Seaber AV . The achilles functional index. JOR Spine. 1992;10:398‐404. 10.1002/jor.1100100313 1569503

[86] Mahler F , Fritschy D . Partial and complete ruptures of the achilles tendon and local corticosteroid injections. Br J Sports Med. 1992;26:7‐14. 1600461 10.1136/bjsm.26.1.7 PMC1478973

[87] Muto T , Kokubu T , Mifune Y , et al. Temporary inductions of matrix metalloprotease‐3 (mmp‐3) expression and cell apoptosis are associated with tendon degeneration or rupture after corticosteroid injection. J Orthop Res. 2014;32:1297‐1304. 24985902 10.1002/jor.22681

[88] Bi Y , Ehirchiou D , Kilts TM , et al. Identification of tendon stem/progenitor cells and the role of the extracellular matrix in their niche. Nat Med. 2007;13:1219‐1227. 17828274 10.1038/nm1630

[89] Huang Z , Yin Z , Xu J , et al. Tendon stem/progenitor cell subpopulations and their implications in tendon biology. Front Cell Dev Biol. 2021;9:631272. 33681210 10.3389/fcell.2021.631272 PMC7930382

[90] Kohler J , Popov C , Klotz B , et al. Uncovering the cellular and molecular changes in tendon stem/progenitor cells attributed to tendon aging and degeneration. Aging Cell. 2013;12:988‐999. 23826660 10.1111/acel.12124 PMC4225469

[91] Komatsu I , Wang JH , Iwasaki K , Shimizu T , Okano T . The effect of tendon stem/progenitor cell (tsc) sheet on the early tendon healing in a rat achilles tendon injury model. Acta Biomater. 2016;42:136‐146. 27329787 10.1016/j.actbio.2016.06.026

[92] Lee CH , Lee FY , Tarafder S , et al. Harnessing endogenous stem/progenitor cells for tendon regeneration. J Clin Invest. 2015;125:2690‐2701. 26053662 10.1172/JCI81589 PMC4563693

[93] Qin S , Wang W , Liu Z , et al. Fibrochondrogenic differentiation potential of tendon‐derived stem/progenitor cells from human patellar tendon. J Orthop Translat. 2020;22:101‐108. 32440505 10.1016/j.jot.2019.08.006 PMC7231964

[94] Zhang X , Lin YC , Rui YF , et al. Therapeutic roles of tendon stem/progenitor cells in tendinopathy. Stem Cells Int. 2016;2016:4076578‐4076514. 27195010 10.1155/2016/4076578 PMC4853952

[95] Zhou Z , Akinbiyi T , Xu L , et al. Tendon‐derived stem/progenitor cell aging: defective self‐renewal and altered fate. Aging Cell. 2010;9:911‐915. 20569237 10.1111/j.1474-9726.2010.00598.x PMC2944918

[96] Li C , Mills Z , Zheng Z . Novel cell sources for bone regeneration. MedComm. 2021;2:145‐174. 34766140 10.1002/mco2.51 PMC8491221

[97] Yoon JH , Halper J . Tendon proteoglycans: biochemistry and function. J Musculoskelet Neuronal Interact. 2005;5:22‐34. 15788868

[98] Zheng Z , Jian J , Zhang X , et al. Reprogramming of human fibroblasts into multipotent cells with a single ecm proteoglycan, fibromodulin. Biomaterials. 2012;33:5821‐5831. 22622142 10.1016/j.biomaterials.2012.04.049

[99] Zheng Z , Jian J , Velasco O , et al. Fibromodulin enhances angiogenesis during cutaneous wound healing. Plast Reconstr Surg Glob Open. 2014;2:e275. 25587509 10.1097/GOX.0000000000000243 PMC4292257

[100] Iozzo RV , Schaefer L . Proteoglycan form and function: a comprehensive nomenclature of proteoglycans. Matrix Biol. 2015;42:11‐55. 25701227 10.1016/j.matbio.2015.02.003 PMC4859157

[101] Iozzo RV , Goldoni S , Berendsen AD , Young MF . The extracellular matrix: an overview. In: Mecham RP , ed. Small Leucine‐Rich Proteoglycans. 1st ed. Springer; 2011. 10.1007/978-3-642-16555-9

[102] Corsi A , Xu T , Chen XD , et al. Phenotypic effects of biglycan deficiency are linked to collagen fibril abnormalities, are synergized by decorin deficiency, and mimic ehlers‐danlos‐like changes in bone and other connective tissues. J Bone Miner Res. 2002;17:1180‐1189. 12102052 10.1359/jbmr.2002.17.7.1180

[103] Kilts T , Ameye L , Syed‐Picard F , et al. Potential roles for the small leucine‐rich proteoglycans biglycan and fibromodulin in ectopic ossification of tendon induced by exercise and in modulating rotarod performance. Scand J Med Sci Sports. 2009;19:536‐546. 19422643 10.1111/j.1600-0838.2009.00909.x PMC2741003

[104] Ruoslahti E . Proteoglycans in cell regulation. J Biol Chem. 1989;264:13369‐13372. 2668264

[105] Iozzo RV . Matrix proteoglycans: from molecular design to cellular function. Annu Rev Biochem. 1998;67:609‐652. 9759499 10.1146/annurev.biochem.67.1.609

[106] Delalande A , Gosselin MP , Suwalski A , et al. Enhanced achilles tendon healing by fibromodulin gene transfer. Nanomedicine. 2015;11:1735‐1744. 26048315 10.1016/j.nano.2015.05.004

[107] Goswami R , Subramanian G , Silayeva L , et al. Gene therapy leaves a vicious cycle. Front Oncol. 2019;9:297. 31069169 10.3389/fonc.2019.00297 PMC6491712

[108] Kaiser J . How safe is a popular gene therapy vector? Science. 2020;367:131. 31919200 10.1126/science.367.6474.131

[109] Rothe M , Schambach A , Biasco L . Safety of gene therapy: new insights to a puzzling case. Curr Gene Ther. 2014;14:429‐436. 25245088 10.2174/1566523214666140918110905

[110] Black S. How one mammalian DNA polymerase challenges the central dogma of biology. The Science Advisory Board 2021; Accessed June 17, 2021. Available from https://www.scienceboard.net/index.aspx?sec=ser&sub=def&pag=dis&ItemID=2839.

[111] Chandramouly G , Zhao J , McDevitt S , et al. Poltheta reverse transcribes rna and promotes rna‐templated DNA repair. Sci Adv. 2021;7:eabf1771. 34117057 10.1126/sciadv.abf1771 PMC8195485

[112] Kafri M , Metzl‐Raz E , Jona G , Barkai N . The cost of protein production. Cell Rep. 2016;14:22‐31. 26725116 10.1016/j.celrep.2015.12.015 PMC4709330

[113] Parekh M , Ali A , Ali Z , et al. Microbioreactor for lower cost and faster optimisation of protein production. Analyst. 2020;145:6148‐6161. 32869772 10.1039/d0an01266a

[114] Kram V , Jani P , Kilts TM , Li L , Chu EY , Young MF . Opg‐fc treatment partially rescues low bone mass phenotype in mature bgn/fmod deficient mice but is deleterious to the young mouse skeleton. J Struct Biol. 2020;212:107627. 32950603 10.1016/j.jsb.2020.107627 PMC7744403

[115] Pourhanifeh MH , Mohammadi R , Noruzi S , et al. The role of fibromodulin in cancer pathogenesis: implications for diagnosis and therapy. Cancer Cell Int. 2019;19:157. 31198406 10.1186/s12935-019-0870-6 PMC6558739

[116] Menon A , Pettinari L , Martinelli C , et al. New insights in extracellular matrix remodeling and collagen turnover related pathways in cultured human tenocytes after ciprofloxacin administration. Muscles Ligaments Tendons J. 2013;3:122‐131. 24367771 PMC3838320

[117] Tsai WC , Hsu CC , Pang JH , Lin MS , Chen YH , Liang FC . Low‐level laser irradiation stimulates tenocyte migration with up‐regulation of dynamin ii expression. PLoS One. 2012;7:e38235. 22666495 10.1371/journal.pone.0038235 PMC3364209

[118] Zhang B , Luo Q , Sun J , et al. Mgf enhances tenocyte invasion through mmp‐2 activity via the fak‐erk1/2 pathway. Wound Repair Regen. 2015;23:394‐402. 25847391 10.1111/wrr.12293

[119] Zhang B , Luo Q , Mao X , et al. A synthetic mechano‐growth factor e peptide promotes rat tenocyte migration by lessening cell stiffness and increasing f‐Actin formation via the fak‐erk1/2 signaling pathway. Exp Cell Res. 2014;322:208‐216. 24434354 10.1016/j.yexcr.2014.01.005

[120] Schaeffer D , Somarelli JA , Hanna G , Palmer GM , Garcia‐Blanco MA . Cellular migration and invasion uncoupled: increased migration is not an inexorable consequence of epithelial‐to‐mesenchymal transition. Mol Cell Biol. 2014;34:3486‐3499. 25002532 10.1128/MCB.00694-14 PMC4135620

[121] Banes AJ , Horesovsky G , Larson C , et al. Mechanical load stimulates expression of novel genes in vivo and in vitro in avian flexor tendon cells. Osteoarthr Cartil. 1999;7:141‐153. 10.1053/joca.1998.0169 10367022

[122] Benjamin M , Ralphs JR . The cell and developmental biology of tendons and ligaments. Int Rev Cytol. 2000;196:85‐130. 10730214 10.1016/s0074-7696(00)96003-0

[123] Randelli F , Menon A , Giai Via A , et al. Effect of a collagen‐based compound on morpho‐functional properties of cultured human tenocytes. Cell. 2018;7:246. 10.3390/cells7120246 PMC6316559 30563214

[124] Chakravarti S . Functions of lumican and fibromodulin: lessons from knockout mice. Glycoconj J. 2002;19:287‐293. 12975607 10.1023/A:1025348417078

[125] Chen S , Birk DE . The regulatory roles of small leucine‐rich proteoglycans in extracellular matrix assembly. FEBS J. 2013;280:2120‐2137. 23331954 10.1111/febs.12136 PMC3651807

[126] Snedeker JG , Foolen J . Tendon injury and repair ‐ a perspective on the basic mechanisms of tendon disease and future clinical therapy. Acta Biomater. 2017;63:18‐36. 28867648 10.1016/j.actbio.2017.08.032

[127] Khunmanee S , Jeong Y , Park H . Crosslinking method of hyaluronic‐based hydrogel for biomedical applications. J Tissue Eng. 2017;8:2041731417726464. 28912946 10.1177/2041731417726464 PMC5590699

[128] Vigetti D , Karousou E , Viola M , Deleonibus S , de Luca G , Passi A . Hyaluronan: biosynthesis and signaling. Biochim Biophys Acta. 2014;1840:2452‐2459. 24513306 10.1016/j.bbagen.2014.02.001

[129] Pascual‐Garrido C , Rodriguez‐Fontan F , Aisenbrey EA , et al. Current and novel injectable hydrogels to treat focal chondral lesions: properties and applicability. J Orthop Res. 2018;36:64‐75. 28975658 10.1002/jor.23760 PMC5839960

[130] Bowman S , Awad ME , Hamrick MW , Hunter M , Fulzele S . Recent advances in hyaluronic acid based therapy for osteoarthritis. Clin Transl Med. 2018;7:6. 29450666 10.1186/s40169-017-0180-3 PMC5814393

[131] Yoshioka K , Yasuda Y , Kisukeda T , Nodera R , Tanaka Y , Miyamoto K . Pharmacological effects of novel cross‐linked hyaluronate, gel‐200, in experimental animal models of osteoarthritis and human cell lines. Osteoarthr Cartil. 2014;22:879‐887. 10.1016/j.joca.2014.04.019 24792209

[132] Evrova O , Kellenberger D , Calcagni M , Vogel V , Buschmann J . Supporting cell‐based tendon therapy: effect of pdgf‐bb and ascorbic acid on rabbit achilles tenocytes in vitro. Int J Mol Sci. 2020;21:458. 31936891 10.3390/ijms21020458 PMC7014238

[133] Wang Y , He G , Tang H , et al. Aspirin inhibits inflammation and scar formation in the injury tendon healing through regulating jnk/stat‐3 signalling pathway. Cell Prolif. 2019;52:e12650. 31225686 10.1111/cpr.12650 PMC6668964

[134] Zheng Z , Lee KS , Zhang X , et al. Fibromodulin‐deficiency alters temporospatial expression patterns of transforming growth factor‐beta ligands and receptors during adult mouse skin wound healing. PLoS One. 2014;9:e90817. 24603701 10.1371/journal.pone.0090817 PMC3948369

[135] Cheng W , Zhang J , Liu J , Yu Z . Granular hydrogels for 3d bioprinting applications. View. 2020;1:20200060.

[136] Sebastin SJ , Ho A , Karjalainen T , Chung KC . History and evolution of the kessler repair. J Hand Surg Am. 2013;38:552‐561. 23395342 10.1016/j.jhsa.2012.11.033 PMC4815901

[137] Wang Y , Jin S , Luo D , et al. Functional regeneration and repair of tendons using biomimetic scaffolds loaded with recombinant periostin. Nat Commun. 2021;12:1293. 33637721 10.1038/s41467-021-21545-1 PMC7910464

[138] Khayyeri H , Hammerman M , Turunen MJ , et al. Diminishing effects of mechanical loading over time during rat achilles tendon healing. PLoS One. 2020;15:e0236681. 33315857 10.1371/journal.pone.0236681 PMC7735574

[139] Dietrich‐Zagonel F , Hammerman M , Bernhardsson M , Eliasson P . Effect of storage and preconditioning of healing rat achilles tendon on structural and mechanical properties. Sci Rep. 2021;11:958. 33441859 10.1038/s41598-020-80299-w PMC7806936

[140] de Medinaceli L , Freed WJ , Wyatt RJ . An index of the functional condition of rat sciatic nerve based on measurements made from walking tracks. Exp Neurol. 1982;77:634‐643. 7117467 10.1016/0014-4886(82)90234-5

[141] Benjamin DJ , Berger JO , Johannesson M , et al. Redefine statistical significance. *Nat* . Hum Behav. 2018;2:6‐10. 10.1038/s41562-017-0189-z 30980045

