# Supplementary material for: A novel injectable fibromodulin‐releasing granular hydrogel for tendon healing and functional recovery
Source: Bioeng Transl Med. 2022 Jul 14;8(1):e10355. doi: 10.1002/btm2.10355 (PMC9842059; doi:10.1002/btm2.10355)
Supplement: Supplementary file 1 — Appendix S1 Supporting Information [file BTM2-8-e10355-s004.docx]

**Supplementary Information**

**Supplemental description of Materials and Methods**

**1. Fibromodulin (FMOD) production**

cDNA of a human FMOD transcript (Genbank assessor number: NM_002023) was subcloned into a commercially available vector pSecTag1A (Thermo Fisher Scientific, Waltham, MA, USA) with a C-terminal His-tag, and then transfected into CHO-K1 cells (ATCC CCL-61; ATCC Manassas, VA, USA).^1^ After establishing a stable FMOD-expression clone, the FMOD was produced and purified by a contract research organization, GenScript (Piscataway, NJ, USA). Briefly, a stable human recombinant FMOD-expressing CHO-K1 cell line was cultured in 1 L of serum-free Freestyle CHO Expression Medium (Thermo Fisher Scientific, CA, USA) at 37℃ with 5% CO_2_ in an Erlenmeyer flask. The cell culture supernatant was harvested after 10 days for purification with HiTrap IMAC HP, 1-mL column (GE Healthcare, Uppsala, Sweden). The fractions from a 100 mM imidazole elution were collected and dialyzed against 20 mM phosphate-buffered saline (PBS), pH 7.4. Next, the sample with low conductivity was loaded onto HiTrap Q HP 1-mL column (GE Healthcare Uppsala, Sweden) for further purification. FMOD was then purified under non-reducing conditions, dialyzed again, and then subjected to lyophilization. The purity of the FMOD product was 85%. FMOD was reconstituted in PBS and then underwent sterilization through a 0.22-μm filter (Thermo Fisher Scientific) before usage.

**2. Cell proliferation assay**

Tenocytes were seeded into a 96-well cell culture plate at a density of 2×10^3^ cells per well. Since the serum is a known source of multiple growth factors,^2^ serum starvation was carried out to standardize the cell cycle phase and eliminate the influence of growth factor persistence for an accurate assessment. After a 16-hour serum starvation, cells were treated with 100 μL fresh medium containing gradient FMOD without serum. After a 72-hour incubation, cell proliferation was measured by the Vybrant MTT Cell Proliferation Assay Kit (Thermo Fisher Scientific).

**3. Cell migration assay**

Cell migration was assessed by wound scratch assay.^3^ Tenocytes were seeded into 6-well tissue culture plates until confluence. After 16 h serum starvation, 1 mm width ‘scratch’ wounds were created by scraping confluent cell monolayers using a sterile pipette tip. To accurately assess cellular migration, each scratch was examined immediately after scraping under a microscope. Only the scratches with a width of 1 ± 0.1 mm were used for further investigation. The wounded monolayer was washed three times with PBS to remove dead cells prior to a 22-hour incubation in 2 mL treatment medium [Tenocyte Growth Medium w/o serum + 800 nM (47.2 μg mL^-1^) FMOD]. Photographs were taken immediately after scratching and 22-hour post-scratching by using an Olympus CellSens standard-microscope imaging software (Olympus America Inc., PA, USA). Migration was quantified by measuring the average wound gaps between the wound edges before and after treatment by using the same software.

**4. Cell invasion assay**

Cell invasion assays were performed in 24-well tissue culture plates using Fluoroblok Cell Culture Inserts with 8-μm pore size Fluorescence Blocking PET track-etched membranes (Corning, NY, USA).^1;4;5^ The upper surface of the inserts was coated with 100 μL 0.5 mg mL^-1^ collagen matrices, rinsed with 83.3 ml tenocyte growth medium (ZenBio, Inc, Durham, NC, USA), and placed into 24-well tissue culture plates containing 500 μL treatment medium (as described above). After a 16-hour serum starvation, 1×10^5^ tenocytes in 100 μL treatment medium were added to each insert chamber and allowed to invade toward the underside of the membrane for 22 hours. Cells that invaded were fixed and stained with 0.4 mg mL^-1^ 4’, 6-diamino-2-phenlindole (DAPI; Millipore Sigma, Steinheim, Germany) before counting. Olympus CellSens standard-microscope imaging software (Olympus America Inc., PA, USA) was used to take images, and Image J [1.53a; National Institutes of Health, USA] was used to count invaded cells.

**5. Cellular gene expression assay**

Tenocytes were seeded at a density of 1×10^5^ cells per dish on 10-cm tissue culture dishes. After a 16-hour serum starvation, cells were incubated with treatment medium (as described above) for 0, 2, 6, 24, 48 and 72 hours. RNAs were extracted using RNeasy Mini Kit (Qiagen, Hilden, Germany) with DNase (Qiagen) treatment, followed by reverse transcription with SuperScript III First-Strand Synthesis System for RT-PCR (Thermo Fisher Scientific). Quantitative real-time PCR (qRT-PCR) was performed on the QuantStudio 3 Real-Time PCR system (Thermo Fisher Scientific) according to the manufacturer’s protocol. The primers and probes used in this study are listed in Table S1. Concomitant glyceraldehyde-3-phosphate dehydrogenase (GAPDH) was performed in separate tubes, as a house-keeping standard. Three different cDNA templates were tested in duplicate.^6-8^ Relative gene expression was normalized to the initial pre-treatment values and analyzed with the _△△_C_T_ method.^1;7;9^

**6. Protein extraction and Western blotting**

Tenocytes were seeded at a density of 1×10^5^ cells per dish on 10-cm tissue culture dishes. After a 16-hour serum starvation, cells were incubated with treatment medium (as described above) up to 72 h. Cells were lysed using Pierce RIPA buffer (Thermo Fisher Scientific) supplied with Halt Protease and Phosphatase Inhibitor Cocktail (Thermo Fisher Scientific) for Western blotting.^6;10^ Antibodies used in this study are listed in Table S2. Western blotting images were captured by ChemiDoc XRS+ system (Bio-Rad Laboratories, Inc, USA) and quantified by Image J. The relative densitometry units were semi-quantified and normalized to the initial pre-treatment values.

**7. Scanning electron microscope (SEM)**

An SEM (FEI NOVA 230, FEI Company) coped with an LVD detector was used for architecture determination of the lyophilized hydrogels at an accelerating voltage of 10 KV.^11^

**8. Mechanical property examination**

The storage modulus and loss modulus obtained from frequency sweep are important for the tissue engineering application as they are capable of revealing the linear equilibrium modulus.^12;13^ Bulk hydrogel was cut into a cubic form at the size of 10 × 10 × 10 mm (L×W×H) and clamped between two parallel-plate compression clamps of a Dynamic Mechanical Analyzer (DMA, Q800; TA instruments, Inc., DE, USA) for the mechanical properties testing. Particularly, the top clamp was gently placed on the hydrogel without force application before turning on the equipment. The storage modulus and loss modulus of the hydrogel were measured at a frequency of 0.1, 1, 10, 20, and 30 Hz where a linear viscoelastic property was documented.^14^

**9. Swelling ratio assessment**

100 μL distilled deionized water was firstly dripped on 3 mg lyophilized granular hydrogel (gHA-hydrogel) with gentle mixing. The reconstituted hydrogel suspension was quickly transferred onto an 8-μm pore size Transwell Insert (Corning), and then put into a 24-well plate containing 1.5 mL PBS. Incubated at 37℃, the hydrogel was weighted in a 24-hour frame. The swelling ratio of HA hydrogel was calculated based on Equation 1:

Swelling ratio = w_t_/ w_0_ × 100% [Equation 1]

Here, the w_t_ and w_0_ represent the hydrogel weight at the time points “t” in incubation and

its initial weight, respectively.^15^

**10. Viscosity measurement**

1 mL distilled deionized water-reconstituted gHA-hydrogels was placed on the bottom plate of a DV-I digital viscometer (Ametek Brookfield, USA) for viscosities testing.^16^ Speed, % torque, and viscosity were documented. Both the bottom plate and the spindle were rinsed with water and wiped with Kimwipe (Kimtech, USA) between different samples measurements to ensure they are clean and dry for the next sample.

**11. FMOD distribution and release analysis**

Considering the molecular weight of FMOD is 59 kDa,^17^ FMOD release from the reconstituted gHA-hydrogel was profiled with a 1,000 kDa dialysis bag (Biotech CE Trial Kit, Spectrum Laboratories, Inc., CA, USA). Briefly, 5 mg lyophilized gHA-hydrogel was reconstituted with 500 μL 6 mg mL^-1^ FMOD and transferred into the dialysis bag, which was then clamped in the ends and placed into a beaker containing 100 mL distilled deionized water. At each time point, 100 μL solution was taken out from the beaker while another 100 μL fresh distilled deionized water was added into the beaker to keep a consistent volume during the entire 8-day testing period. The obtained drug release was plotted according to zero-order model (cumulative % FMOD released vs. time), first-order model [log_10_(cumulative % FMOD remaining) vs. time], Higuchi model (cumulative % FMOD released vs. square root of time), Hixson-Crowell model (cubic root of cumulative % FMOD released vs. time), and Korsmeyer-Peppas model [log_10_(cumulative % FMOD released) vs. log_10_(time)] equations.^18-20^

The distribution of FMOD in the reconstituted gHA-hydrogel was assessed by the one-way diffusion test.^21;22^ Briefly, 5 mg lyophilized gHA-hydrogel was reconstituted with 500 μL 6 mg mL^-1^ FMOD for 15 min, transferred into 8-μm pore size Transwell Insert (Corning) in a 24-well cell culture plate containing 1 mL distilled deionized water into the lower chamber. At each time point, 100 μL solution was taken out from lower chamber for FMOD concentration measurement, while another 100 μL fresh distilled deionized water was added into the lower chamber to keep a consistent volume during the entire 25-day testing period. BCA Protein Assay Kit (Thermo Fisher Scientific) was used for protein quantification with a microplate spectrophotometer (US BioTek Laboratories, WK, US) and Gen5 software (Version 2.04; US BioTek Laboratories, WK, US). Meanwhile, linear slope analysis identified different diffusion phases in the fractional diffusion graph where the largest R^2^ values were obtained in the linear progression.^23;24^

**12. Injectability testing**

The injectability of the FMOD/gHA-hydrogel was presented by its syringeability. 1 mL reconstituted gHA-hydrogel with 6 mg mL^-1^ FMOD was loaded into a Tuberculin Syringe (BD Biosciences, NJ, USA) with a 27 Gauge × 1/2 inch detachable needle (BD Biosciences). The syringeability was tested by gently pushing the plunge (Video S1).

**Supplemental tables**

**Table S1. Primers and probes for qRT-PCR**

*Taqman^@^ Gene Expression Assays purchased from Thermo Fisher Scientific*

| Gene name | Catalog number |
| --- | --- |
| *Mmp2* | Rn01538170_m1 |
| *Mmp3* | Rn00591740_m1 |
| *Mmp9* | Rn00579162_m1 |
| *Mmp14* | Rn00579172_m1 |
| *Col1𝛼1* | Rn01463848_m1 |
| *Col3𝛼1* | Rn01437681_m1 |
| *Lox* | Rn01491829_m1 |
| *Gapdh* | Rn99999916_s1 |

**Table S2. Primary antibodies for western blotting and immunofluorescence staining**

| Antigen | Company | Catalog number | Reference |
| --- | --- | --- | --- |
| GAPDH | Gene TEX | GTX100118 | Kuo, et al., 2017^25^ |
| MMP3 | Abcam Inc. | ab53015 | Hafez et al., 2018^26^ |
| MMP9 | Abcam Inc. | ab76003 | Jiang, et al., 2020^27^ |
| LOX | Abcam Inc. | ab174316 | Ma, et al., 2021^28^ |
| Vimentin | Abcam Inc. | ab8978 | Roulois et al., 2016^29^ |
| Tenomodulin | Abcam Inc. | ab203676 | Li, et al., 2019^30^ |


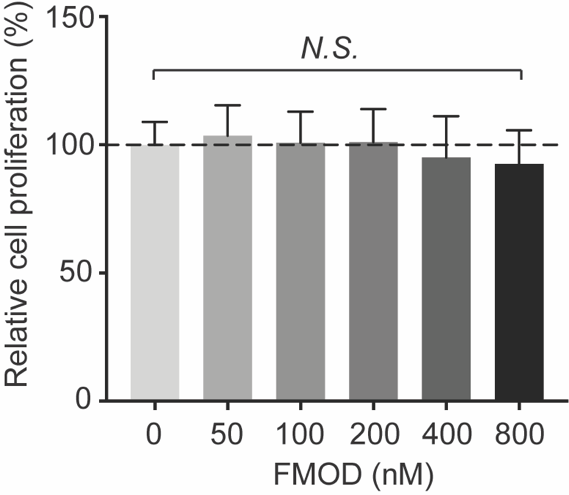


**Fig. S1.** **FMOD did not significantly affect tenocyte proliferation *in vitro*.**

Data are presented as mean ± SD, *n* = 6. One-way ANOVA was used for statistical analyses. *N.S*., no statistical significance, *P* > 0.05.


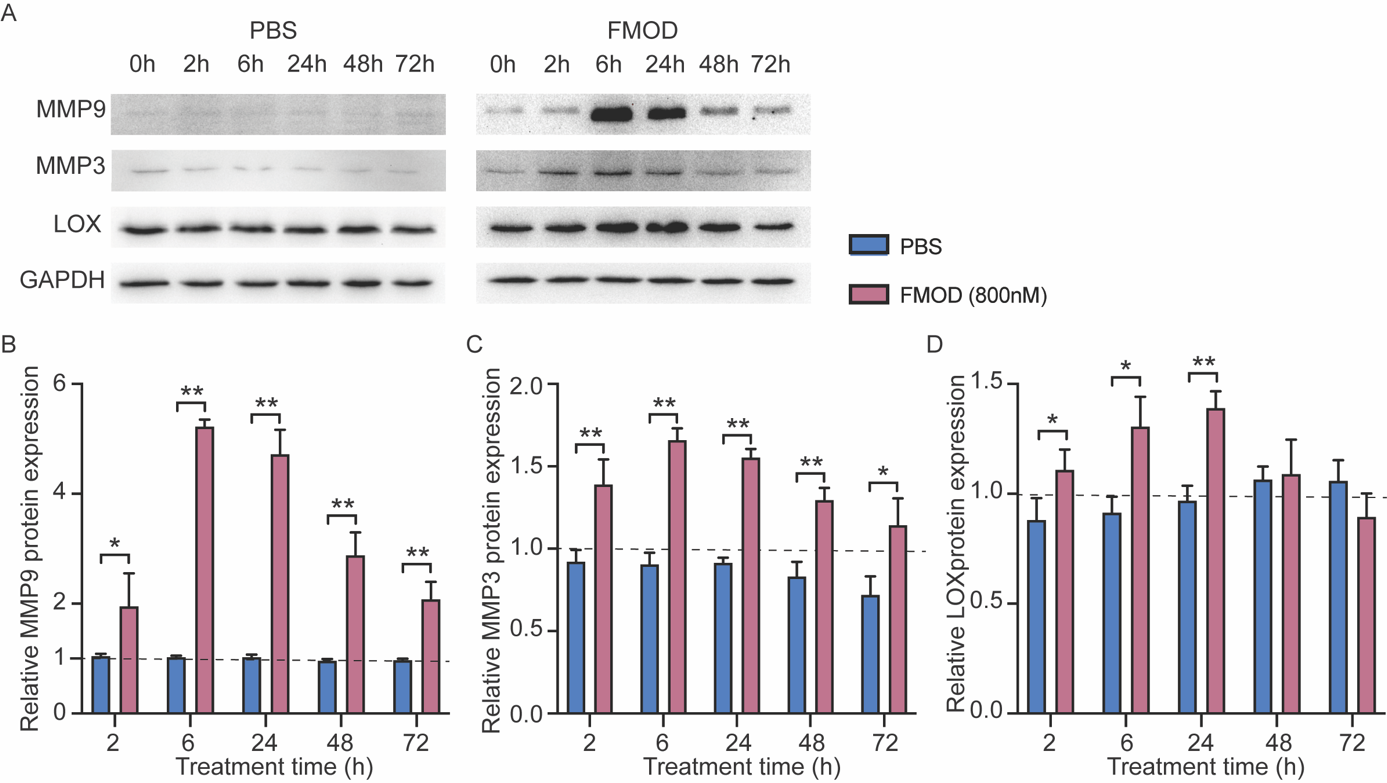


**Fig. S2.** **FMOD significantly enhanced the protein expression level of MMP3, MMP9, and LOX.**

Western-blotting (A) bands of MMP9 (B), MMP3 (C), and LOX (C) were semi-quantified against the responsive GAPDH bands and normalized to the 0 h values. Data are shown as mean ± SD, *n* = 3. Two-sample *t*-tests were used for statistical analyses. * *P* < 0.05; ** *P* < 0.005*.*


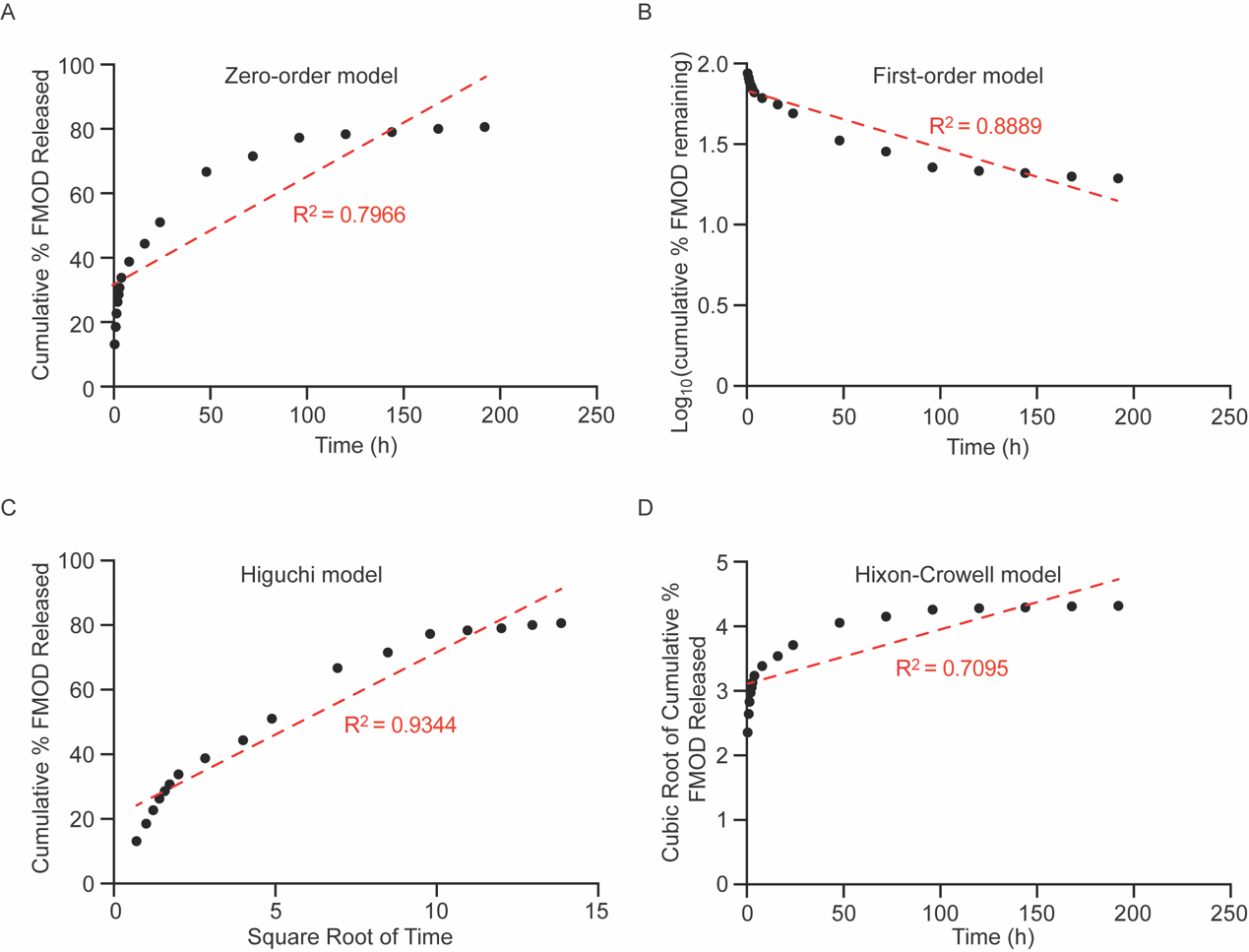


**Fig. S3** The release profile of FMOD from the reconstituted gHA-hydrogel was fitted in zero-order (A), first-order (B), Higuchi (C), and Hixon-Crowell (D) kinetic models.


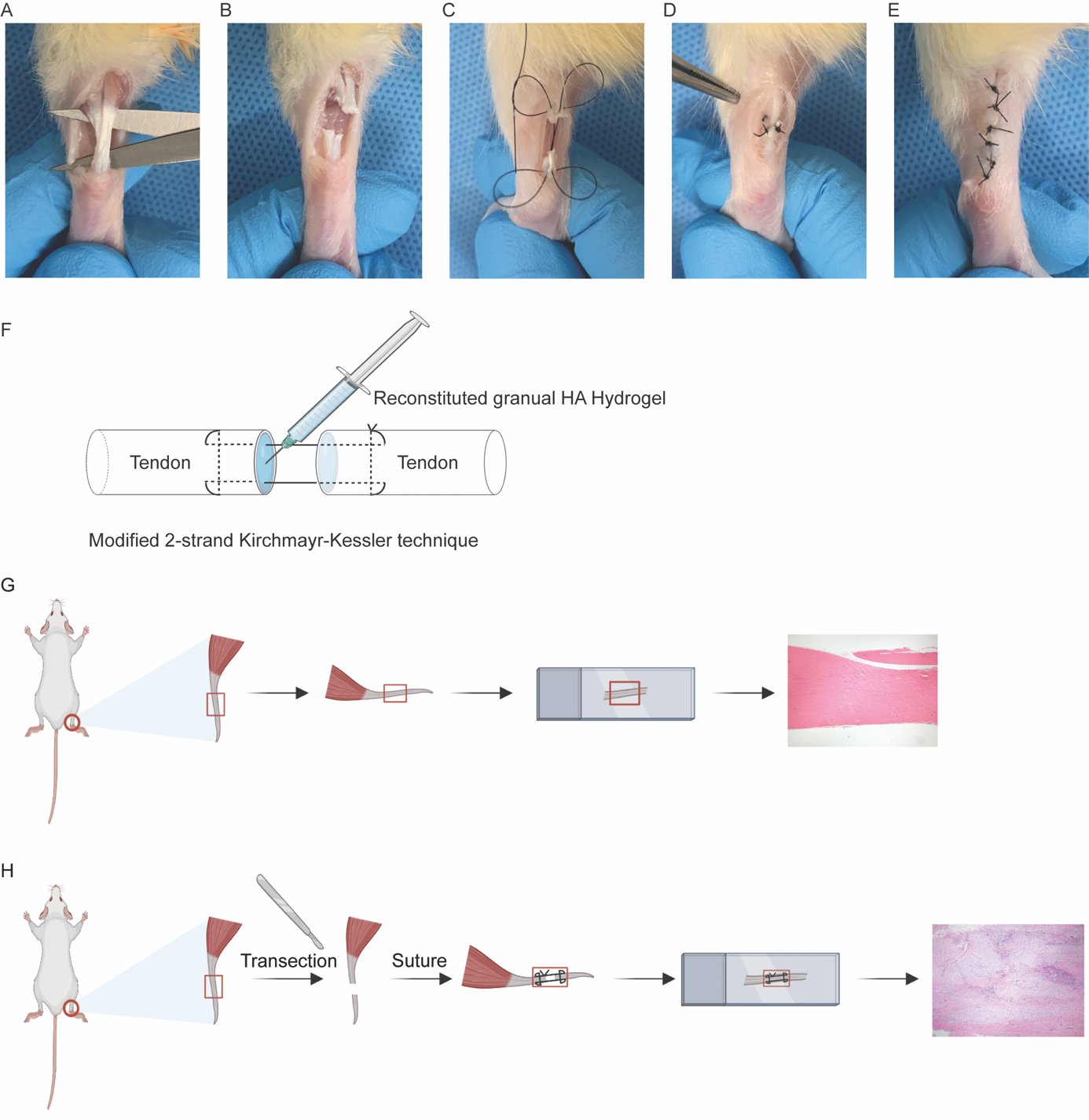


**Fig. S4. The operation processes of rat tendon wounds model.**

A longitudinal skin incision was made to expose the Achilles tendon of adult female Sprague-Dawley rats (A). Then, the Achilles tendon was transected in the middle (B). After placing the reconstituted granular HA hydrogel on each wound edge, the wounded tendon was sutured with 5-0 Nylon by using the Modified 2-strand Kirchmayr-Kessler technique (C, D). Last, the skin incision was closed in layers with an interrupted suture (E). An illustration of applying 5 μL reconstituted granular HA hydrogel on each wound edge by using a syringe is also shown (F). The whole length of the Achilles tendon was harvested and sectioned 21 days after the operation. Both the unwounded tendon (G) and wounded tendon (H) were placed in the same direction on the slides for histological assessment: the end connected to the calcaneus originated right in the photos while the end connected to the calf muscle was originated left.


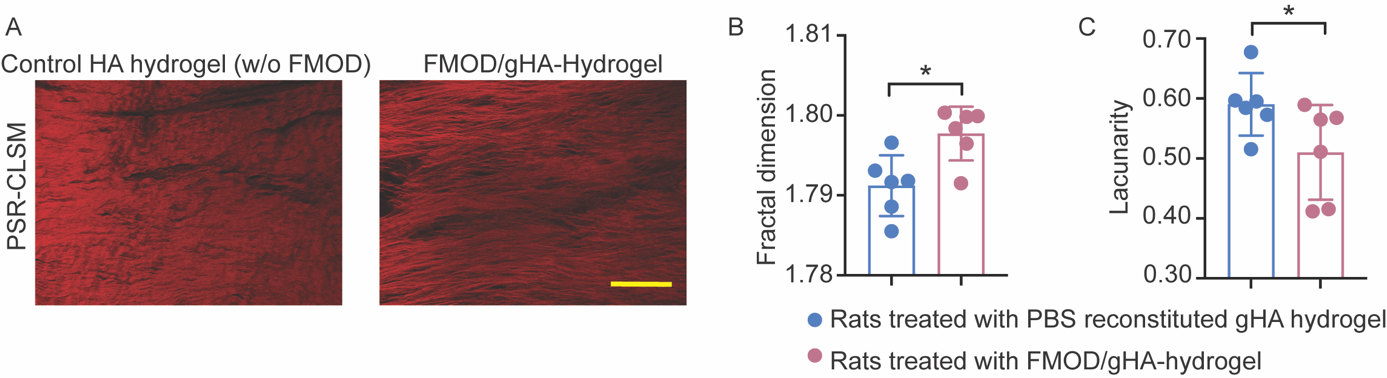


**Fig. S5. Topological analyses were also used to assess the collagen architecture of the wounded tendons.**

PSR stained tissues were documented by CLAM (A), and then evaluated by Fractal dimension (B) and Lacunarity (C). Scale bar = 25 μm. Data are shown as mean ± SD, *n* = 6. Two-sample *t*-tests were used for statistical analyses. * *P* < 0.05*.*


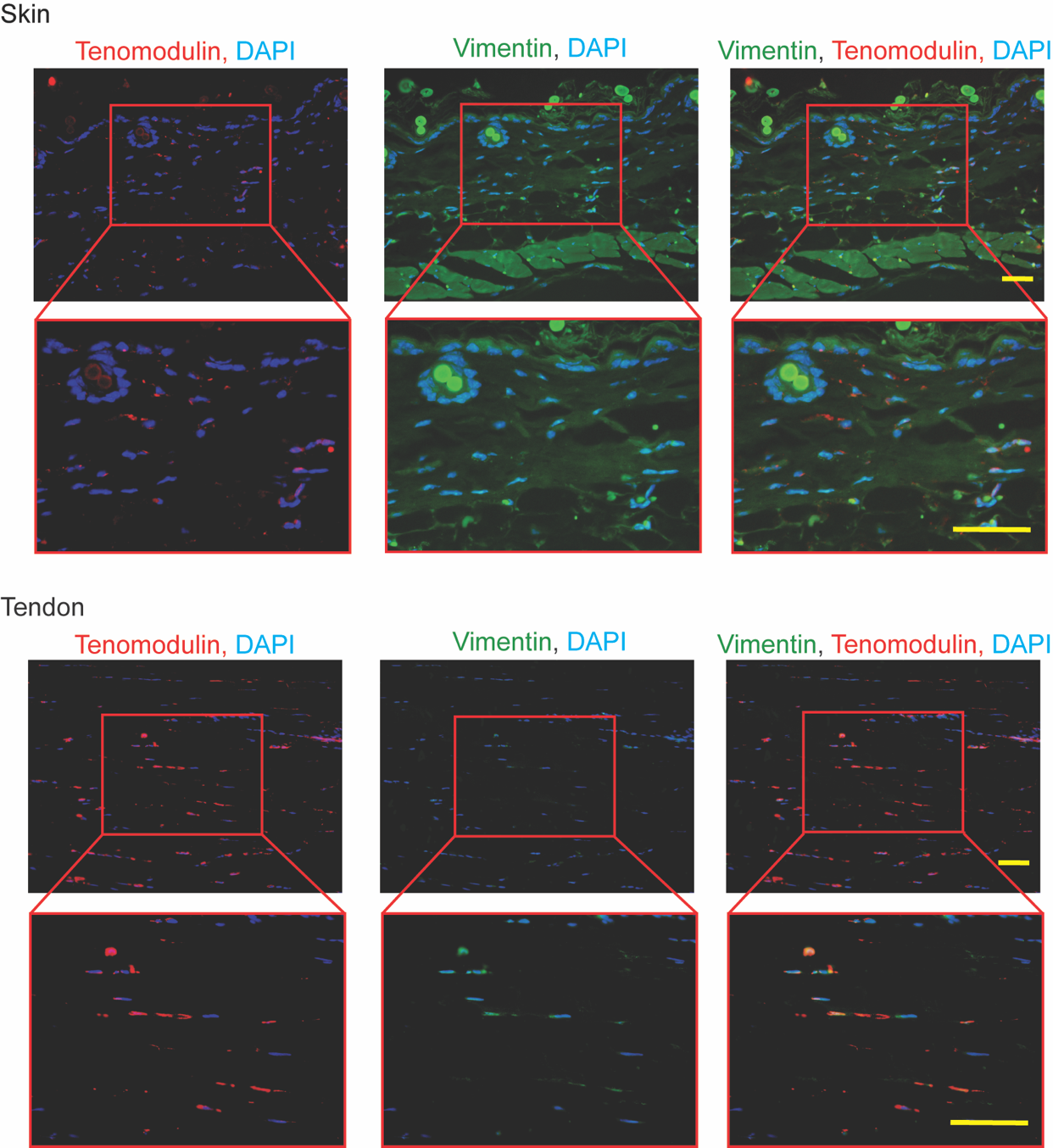


**Fig. S6. Tenomodulin (Tnmd) is a specific marker of mature tenocytes.**

Skin tissue was used as a negative control. Vimentin was used to stain non-tenogenic fibroblasts, while DAPI was used for nuclei counterstaining. Scale bar = 100 μm, respectively.

**References for Supplemental Materials**

1. Zheng Z, Nguyen C, Zhang X, et al. Delayed wound closure in fibromodulin-deficient mice is associated with increased tgf-beta3 signaling. *J Invest Dermatol*. 2011;131:769-778.

2. Lin TH, Shao YY, Chan SY, et al. High serum transforming growth factor-beta1 levels predict outcome in hepatocellular carcinoma patients treated with sorafenib. *Clin Cancer Res*. 2015;21:3678-3684.

3. Xue M, Smith MM, Little CB, et al. Activated protein c mediates a healing phenotype in cultured tenocytes. *J Cell Mol Med*. 2009;13:749-757.

4. Zhang B, Luo Q, Sun J, et al. Mgf enhances tenocyte invasion through mmp-2 activity via the fak-erk1/2 pathway. *Wound Repair Regen*. 2015;23:394-402.

5. Kimmerling KA, McQuilling JP, Staples MC, Mowry KC. Tenocyte cell density, migration, and extracellular matrix deposition with amniotic suspension allograft. *J Orthop Res*. 2019;37:412-420.

6. Zheng Z, Li C, Ha P, et al. Cdkn2b upregulation prevents teratoma formation in multipotent fibromodulin-reprogrammed cells. *J Clin Invest*. 2019;129:3236-3251.

7. Zheng Z, Jian J, Zhang X, et al. Reprogramming of human fibroblasts into multipotent cells with a single ecm proteoglycan, fibromodulin. *Biomaterials*. 2012;33:5821-5831.

8. Li CS, Yang P, Ting K, et al. Fibromodulin reprogrammed cells: A novel cell source for bone regeneration. *Biomaterials*. 2016;83:194-206.

9. Zheng Z, Lee KS, Zhang X, et al. Fibromodulin-deficiency alters temporospatial expression patterns of transforming growth factor-beta ligands and receptors during adult mouse skin wound healing. *PLoS One*. 2014;9:e90817.

10. Zheng Z, James AW, Li C, et al. Fibromodulin reduces scar formation in adult cutaneous wounds by eliciting a fetal-like phenotype. *Signal Transduct Target Ther*. 2017;2:17050.

11. Sun N, Wang T, Yan X. Self-assembled supermolecular hydrogel based on hydroxyethyl cellulose: Formation, in vitro release and bacteriostasis application. *Carbohydr Polym*. 2017;172:49-59.

12. Farnebo S, Woon CY, Schmitt T, et al. Design and characterization of an injectable tendon hydrogel: A novel scaffold for guided tissue regeneration in the musculoskeletal system. *Tissue Eng Part A*. 2014;20:1550-1561.

13. Imai K, Ikoma K, Chen Q, et al. Biomechanical and histological effects of augmented soft tissue mobilization therapy on achilles tendinopathy in a rabbit model. *J Manipulative Physiol Ther*. 2015;38:112-118.

14. Meyvis TKL, Stubbe BG, Van Steenbergen MJ, et al. A comparison between the use of dynamic mechanical analysis and oscillatory shear rheometry for the characterisation of hydrogels. *International Journal of Pharmaceutics*. 2002;244:163-168.

15. Wu F, Pang Y, Liu J. Swelling-strengthening hydrogels by embedding with deformable nanobarriers. *Nat Commun*. 2020;11:4502.

16. Ofori-Kwakye KA, Y.; SAMUEL LUGRIE Kipo. Physicochemical and binding properties of cashew tree gum in metronidazole tablet formulations. *International Journal of Pharmacy and Pharmaceutical Sciences* 2010;2:105-109.

17. D’Souza S. A review of in vitro drug release test methods for nano-sized dosage forms. *Advances in Pharmaceutics*. 2014;2014:304757.

18. Hina Kouser Shaikh RK, S. G. Patil. Mathematical models for drug release characterization: A review. *World J. Pharm. Pharm. Sci*. 2015;4:324-338.

19. Sjoholm E, Mathiyalagan R, Rajan Prakash D, et al. 3d-printed veterinary dosage forms-a comparative study of three semi-solid extrusion 3d printers. *Pharmaceutics*. 2020;12.

20. Dash S, Murthy PN, Nath L, Chowdhury P. Kinetic modeling on drug release from controlled drug delivery systems. *Acta Pol Pharm*. 2010;67:217-223.

21. Domb A, Davidson GWR, Sanders LM. Diffusion of peptides through hydrogel membranes. *J Control Release*. 1990;14:133-144.

22. Hsu HH, Kracht JK, Harder LE, et al. A method for determination and simulation of permeability and diffusion in a 3d tissue model in a membrane insert system for multi-well plates. *J Vis Exp*. 2018.

23. Kopac T, Rucigaj A, Krajnc M. The mutual effect of the crosslinker and biopolymer concentration on the desired hydrogel properties. *Int J Biol Macromol*. 2020;159:557-569.

24. Macha IJ, Ben-Nissan B, Vilchevskaya EN, et al. Drug delivery from polymer-based nanopharmaceuticals-an experimental study complemented by simulations of selected diffusion processes. *Front Bioeng Biotechnol*. 2019;7:37.

25. Kuo YL, Cheng JK, Hou WH, et al. K(+) channel modulatory subunits kchip and dpp participate in kv4-mediated mechanical pain control. *J Neurosci*. 2017;37:4391-4404.

26. Hafez S, Abdelsaid M, Fagan SC, Ergul A. Peroxynitrite-induced tyrosine nitration contributes to matrix metalloprotease-3 activation: Relevance to hyperglycemic ischemic brain injury and tissue plasminogen activator. *Neurochem Res*. 2018;43:259-266.

27. Jiang L, Xu K, Li J, et al. Nesfatin-1 suppresses interleukin-1beta-induced inflammation, apoptosis, and cartilage matrix destruction in chondrocytes and ameliorates osteoarthritis in rats. *Aging (Albany NY)*. 2020;12:1760-1777.

28. Ma M, Wu CJ, Zhang P, et al. N-acetylcysteine maintains penile length and erectile function in bilateral cavernous nerve crush rat model by reducing penile fibrosis. *Asian J Androl*. 2021;23:215-221.

29. Roulois D, Deshayes S, Guilly MN, et al. Characterization of preneoplastic and neoplastic rat mesothelial cell lines: The involvement of tets, dnmts, and 5-hydroxymethylcytosine. *Oncotarget*. 2016;7:34664-34687.

30. Li W, Midgley AC, Bai Y, et al. Subcutaneously engineered autologous extracellular matrix scaffolds with aligned microchannels for enhanced tendon regeneration: Aligned microchannel scaffolds for tendon repair. *Biomaterials*. 2019;224:119488.
